# Rhizosphere-dwelling halophilic archaea: a potential candidate for alleviating salinity-associated stress in agriculture

**DOI:** 10.3389/fmicb.2023.1212349

**Published:** 2023-07-26

**Authors:** Mayur G. Naitam, B. Ramakrishnan, Monendra Grover, Rajeev Kaushik

**Affiliations:** ^1^Division of Microbiology, ICAR-Indian Agricultural Research Institute, New Delhi, India; ^2^Center for Agricultural Bioinformatics, ICAR-Indian Agricultural Statistical Research Institute, New Delhi, India

**Keywords:** halophilic archaea, wheat, salinity alleviation, abiotic stress, plant growth promotion

## Abstract

Salinity is a serious environmental factor that impedes crop growth and drastically reduces yield. This study aimed to investigate the potential of halophilic archaea isolated from the Rann of Kutch to alleviate the negative impact of salinity on crop growth and yield. The halophilic archaea, which demonstrated high tolerance to salinity levels up to 4.5 M, were evaluated for their ability to promote plant growth in both salt-tolerant and salt-susceptible wheat cultivars. Our assessment focused on their capacity to solubilize essential nutrients, including phosphorus (14-61 mg L^−1^), potassium (37-78 mg L^−1^), and zinc (8-17 mg L^−1^), as well as their production of the phytohormone IAA (17.30 to 49.3 μg ml^−1^). To conduct the experiments, five wheat cultivars (two salt-tolerant and three salt-susceptible) were grown in triplicates using soft MS agar tubes (50 ml) and pots containing 10 kg of soil with an electrical conductivity (EC) of 8 dSm^−1^. Data were collected at specific time points: 21 days after sowing (DAS) for the MS agar experiment, 45 DAS for the pot experiment, and at the time of harvest. In the presence of haloarchaea, the inoculated treatments exhibited significant increases in total protein (46%), sugar (27%), and chlorophyll (31%) levels compared to the un-inoculated control. Furthermore, the inoculation led to an elevated accumulation of osmolyte proline (31.51%) and total carbohydrates (27.85%) while substantially reducing the activity of antioxidant enzymes such as SOD, catalase, and peroxidase by 57–76%, respectively. Notably, the inoculated treatments also showed improved plant vegetative growth parameters compared to the un-inoculated treatments. Interestingly, the positive effects of the halophilic archaea were more pronounced in the susceptible wheat cultivars than in the tolerant cultivars. These findings highlight the growth-promoting abilities of the halophilic archaeon *Halolamina pelagica* CDK2 and its potential to mitigate the detrimental effects of salinity. Consequently, further evaluation of this halophilic archaeon under field conditions is warranted to explore its potential use in the development of microbial inoculants.

## 1. Introduction

Salinity is one of the most detrimental kinds of abiotic stress that affects agricultural productivity and food security (Sardar et al., 2023). Soil salinity causes several morphological, physiological, and molecular changes in crop plants which result in decreased growth and productivity. The enhanced production of reactive oxygen species in plants under oxidative stress induced by salinity causes damage to the cell membrane, proteins, lipids, and nucleic acids and triggers programmed cell death (Gill and Tuteja, 2010; Calanca, 2017). Globally, an estimated 20% of arable soil is affected by salinity at the moment, and that number may rise to 50% by 2050 due to a variety of factors, including the lack of effective mitigation practices (Rakshit et al., 2020). Efficient resource management and crop improvement for the development of better cultivars can aid in the reduction of salinity stress. To reduce salinity stress in agriculture, commonly practiced strategies include soil and irrigation management, selecting and breeding salt-tolerant crop varieties, implementing agronomic practices, such as mulching, crop rotation, and intercropping, leaching excess salts, and employing biotechnological interventions to develop and utilize salt-tolerant plant varieties (Ondrasek et al., 2022). Given the time-consuming and expensive nature of current strategies, it is crucial to develop cost-effective and easily adaptable biological methods to manage agricultural stress. These methods can have direct implications in the near future. Microorganisms could play an important role in this regard if we take advantage of their unique properties. Not only microbial inoculation can help in the control of different stresses, but also it can trigger plant development and production and the improvement of soil fertility (Chaudhary et al., 2023). These include salinity tolerance, the ability to thrive in varying water conditions, production of plant growth-promoting hormones, nutrient cycling, siderophore production, volatile organic compound production, ACC deaminase activity, antioxidant capabilities, and the ability to produce osmolytes. They also interact with other microbes and crops, thereby helping plants withstand multiple stresses simultaneously (Sharma et al., 2021; Suman et al., 2022). In this regard, the ability of bacterial inoculants to alleviate biotic and abiotic stresses in plants is well documented and demonstrated, but the members of the domain Archaea are still under explored (Naitam and Kaushik, 2021).

Prokaryotes from the domain Archaea have long been considered native inhabitants of ecological niches with extreme conditions of temperature, acidity, alkalinity, and salinity (Gubry-Rangin et al., 2010; Naitam and Kaushik, 2021). However, this notion of a scientific community has changed since the advent of next-generation genome sequencing techniques (metagenomics and metatranscriptomics) that have revolutionized the study of microbial diversity (Oren, 2014; Yadav et al., 2017). In recent decades, archaea have also been isolated from a wide range of ecological niches that have normal environmental conditions; these include arable and barren lands, plant rhizosphere, freshwater lakes, sediments, humans, and animals' guts (Kumar et al., 2009; Gubry-Rangin et al., 2010; Yadav et al., 2019; Jung et al., 2020; Akinola and Babalola, 2021; Hu et al., 2021). Archaea also constitute a significant proportion of microbiomes, phytobiomes, and various plant-associated ecosystems (Jung et al., 2020). Archaea can play a potential role in improving crop production and sustainability in arid and semi-arid (Alori et al., 2020). Few studies reported the presence of archaea and their role in improving plant growth. Thaumarchaeota was reported as dominant phyla in the rhizospheric soils of native alpine trees of the Qinghai-Tibetan Plateau along with some unclassified archaeal groups as keystone species. The results suggested that the archaeal community structure in rhizospheric soil is simple as opposed to bulk soils (Zhang et al., 2020). Cultivar-specific variation (rhizospheric effect) in the archaeal community was observed in tomatoes; however, a many-fold reduction in the archaeal community was observed during vertical transmission in the F1 generation (Taffner et al., 2020). The archaeal community in tomato rhizosphere studies was dominated by Thaumarchaeota and Euryarchaeota (Taffner et al., 2020).

Among archaea, the halophilic archaea belonging to the class Halobacteria and phylum Euryarchaeota have significant economic importance due to their wider adaptability to salinity levels for growth (1.5 M NaCl to saturation) (Oren, 2014; Naitam and Kaushik, 2021). Halophilic archaea are natural inhabitants of the hypersaline environment of salt lake and salt evaporation ponds (Oren, 2014, 2019). Halophilic archaea *Haloferax* sp. strain NARS9, isolated from the solar salterns from the coast of Jeddah, Saudi Arabia, ameliorated the cobalt heavy metal stress and improved grain yield in wheat (Hagagy et al., 2023). Halophilic archaea found in the rhizosphere of plants in high-salinity areas can be assessed for their potential to alleviate salinity stress and enhance crop growth and productivity in cultivated regions affected by salinity. In the pursuit of exploring such possibilities, 28 halophilic archaea were isolated and characterized from bulk soil, water bodies, and rhizosphere of wild vegetation in the saline desert of Rann of Kutch, Gujarat, India. The salinity levels in this region ranged from 1.19 to 106.7 dSm-1, and the pH varied from 7.40 to 10.15 (Yadav et al., 2015). Most of the isolates belonged to 16 genera, viz. *Halobacterium, Halococcus, Halolamina, Haladaptatus, Haloarcula, Haloferax, Halogeometricum, Halopenitus, Halosarcina, Halostagnicola, Haloterrigena, Halorubrum, Natrialba, Natrinema, Natronoarchaeum*, and *Natronomonas*. In many of the isolates belonging to these 16 genera, solubilization of inorganic phosphorous was reported (Yadav et al., 2015). Studies on the population dynamics of halophilic archaea in different samples revealed seasonal variation. The highest and lowest diversity and population of archaea were observed in winter and autumn, respectively, whereas some of the archaea were season-specific (Yadav et al., 2019). Furthermore, in lab-scale studies, we observed stimulation of Wheat and Bajara germination upon inoculation of some of these isolates of halophilic archaea in a saline soft agar medium having electrical conductivity (EC) ranging from 6.8 to 8.4 dSm^−1^.

Hence, a hypothesis can be proposed that halophilic archaea inhabiting the rhizosphere of wild vegetation in hypersaline soils of Rann of Kutch, Gujarat, India, may have developed a mutualistic relationship with the plant system. It is likely that they assist in the establishment and growth of the plants by mitigating the detrimental effects of salinity. However, to date, very few significant findings are available which can prove that halophilic archaea can promote plant growth by alleviating the harmful effects of salinity.

Based on soil EC, the saline soils are classified as slightly saline (4.0–8.0 dSm^−1^), moderately saline (8–30 dSm^−1^), and strong saline (>30.0 dSm^−1^) (Gorji et al., 2020). The optimal range of soil EC for the growth of crops ranges from 1 to 2 dSm^−1^, and an increase in soil EC from slightly to strongly saline significantly reduces crop growth and productivity (Sadaty and Nazari, 2021). The EC of soil in different regions of Rann of Kutch varies from 1.19 to 106.7 dSm^−1^ (Yadav et al., 2019). The halophilic archaea in our previous study were isolated from barren soil (EC >75.25 dSm^−1^) and rhizosphere of wild vegetation growing in soil having EC ranging from 4.79 to 34.2 dSm^−1^ (Yadav et al., 2019). In this study, the ability of halophilic archaeal isolates to thrive in moderately saline soil was examined. Additionally, their potential as bioinoculants for enhancing wheat growth was explored through nutrient solubilization, phytohormone production, and mitigation of salinity-induced oxidative stress.

## 2. Materials and methods

### 2.1. Procurement of halophilic archaea and quantification of their growth

In total, 28 halophilic archaea previously isolated from the rhizosphere of wild plants growing in the hypersaline region of the Rann of Kutch, Gujarat, India, were procured from the microbial culture collection of the Division of Microbiology, Indian Agricultural Research Institute, New Delhi, India. All the isolates were cultured in the liquid broth of a modified DSMZ 1184 medium, and their growth was quantified in terms of μg protein ml^−1^. The composition of the DSMZ 1184 growth medium was modified and standardized for culturing of halophilic archaea inhabiting plant rhizosphere. The modified media used in this study was called the halophilic rhizospheric archaea (HRA) medium. Its composition per liter included the following: 1.0 g of sodium pyruvate, 1.0 g of yeast extract, 80.0 g of NaCl, 32.5 g of MgCl_2_.6H_2_O, 50.8 g of MgSO_4_.7H_2_O, 5.0 g of KCl, 0.25 g of NaHCO_3_, 1.0 g of NaNO_3_, 0.8 g of CaCl_2_.2H_2_O, 0.05 g of KH_2_PO_4_, 0.03 g of NH_4_Cl, traces of FeSO_4_.7H_2_O, traces of MnSO_4_.7H_2_O, and 20 g of agar. pH was adjusted to 7.4 with a 1 M Tris base, and the final EC of the media was 119.8 dSm^−1^. The stock solution of NaHCO3 and sodium pyruvate was filter-sterilized and added aseptically to the autoclaved HRA medium. Approximately 1 ml of actively growing inoculum containing 10^8^ CFU ml^−1^ of the respective isolates was inoculated in the 50 ml HRA broth and incubated at 37 °C at 130 rpm. After 10 d of incubation, the 5 ml of culture broth was centrifuged at 7,500 *g* for 10 min, and the pellet was used for quantification of protein by Bradford's method (Marion, 1976). The amount of protein in the culture was extrapolated by comparison with a standard curve prepared using different concentrations of bovine serum albumin (BSA).

### 2.2. Evaluation of PGP attributes

#### 2.2.1. Solubilization of P, K, and Zn

All 28 halophilic archaeal isolates were assessed qualitatively and quantitatively for solubilization of phosphorus (P), potassium (K), and zinc (Zn). The HRA medium was modified by adding 5.0 g L^−1^ tricalcium phosphate to estimate P solubilization (HPS medium), and KCl was replaced with 5.0 g L^−1^ potassium aluminum silicate to estimate K solubilization (HKS medium). Similarly, Zn solubilization was quantified using two sets of HRA medium supplemented with zinc oxide and zinc carbonate, respectively (added at 1.0 gL^−1^) (HZS medium). For qualitative estimation of P, K, and Zn solubilization, 10 μl of log phase cultures were spot-inoculated on respective modified HRA agar and incubated at 37 °C. The formation of the halo zone around colonies was recorded periodically until the 10th day of incubation and the D/D ratio was calculated, i.e., the diameter of the zone of solubilization/colony diameter. The cultures which solubilized the P, K, and Zn with a D/D ratio higher than one were selected for quantifying the extent of their solubilization. For this, 1 ml inoculum (10^8^ CFU ml^−1^) of the selected cultures was inoculated in 50 ml (for P solubilization) and 100 ml (for K and Zn solubilization) of respective HRA broth and incubated at 37 °C at 130 rpm. After 10 d of incubation, the pH of the culture broth was recorded and subjected to centrifugation at 10, 000 *g* for 5 min. The supernatant was used for quantitative estimation of soluble P, K, and Zn by the ascorbic acid method (Yadav et al., 2015), flame photometer (Banerjee and Prasad, 2020), and atomic absorption spectrophotometer (ZEEnit 700 P, Analytik Jena, Gmbh) (Saravanan et al., 2004), respectively. The experiment was performed in triplicates, and un-inoculated control was maintained.

#### 2.2.2. Quantification of indole-3-acetic acid production

The production of indole-3-acetic acid (IAA) by 28 isolates of halophilic archaea was quantified by culturing them in HRA broth supplemented with L-tryptophane (at 500μg ml^−1^). The 50 ml broth was inoculated with 1 ml of actively growing inoculum (10^8^ CFU ml^−1^) and incubated at 37 °C for 10 d at 130 rpm. Following incubation, the culture was centrifuged at 5,500 *g* for 10 min, and 2 ml supernatant was used for spectrophotometric estimation of IAA production by Salkowski's reagent method (Goswami et al., 2014). The concentration of IAA in the samples was inferred by comparison with the standard curve. The un-inoculated medium was used as blank, and the experiment was conducted in triplicates.

### 2.3. Growth and PGPR studies in soil extract medium

#### 2.3.1. Preparation of HSE medium

Plant growth-promoting rhizobacteria (PGPR) play a big role in the mitigation of abiotic stresses by improving the expansion of soil colonization by plant roots. This can be reflected in higher affordability to retain the needed nutrients for plant growth and development (Širić et al., 2022). To determine whether the halophilic archaea can grow in soils having slight (4.0 to 8.0 dsm^−1^) to moderate (8.0 to 30.0 dsm^−1^) salinity, a modified halophilic broth was prepared using soil extract, where the water component was replaced with soil extract. The soil extract was prepared from the soil sample collected from a wheat field (15 cm depth) located at the village Chhapra Khera (29°40′42.3^′′^N and 77°02′50.7^′′^E), Karnal, Haryana, India. The soil was sandy loam in texture with an EC of 6.2 dsm^−1^, pH 8.1, total carbon: 0.48 %, total nitrogen: 0.051%, exchangeable K: 173.61 mg Kg^−1^, and available P: 4.21 mg Kg^−1^. For the preparation of soil extract, 400 g of fine soil was mixed with 1600 ml of tap water and mixed thoroughly to make a uniform solution. The mixture was heated at 121°C for 15 min. After cooling to room temperature, the mixture was filtered through a sterile muslin cloth followed by filter paper (Whatman No. 1). The filtrate was used as a water component in the preparation of halophilic broth. The HRA broth was modified to develop halophilic soil extract (HSE) broth having EC and nutrient mineral composition (gL^−1^) as shown in Table 1. The composition of major mineral ingredients (NaCl, MgCl_2_.6H_2_O, MgSO_4_.7H_2_O, KCl, NaHCO_3_, NaNO_3_, and CaCl_2_.2H_2_O) was subjected to change to achieve desired EC levels, and the composition of remaining minor mineral nutrients (KH_2_PO_4_, NH_4_Cl, FeSO4.7H2O, and MnSO4.7H2O) was kept constant. The medium was denoted as HSE-1 to HSE-9 (EC: 7.13–30.84 dsm^−1^). HRA medium (EC: 119.8 dsm^−1^) was used as a reference control for comparing the growth and PGP properties of selected halophilic archaea. The HSE broth (HSE-1 to HSE-9) was used to quantify the growth and PGP attributes of *H. pelagica* CDK2.

**Table 1:** Composition and salinity levels (EC) of halophilic soil extract (HSE) broth.

**Halophilic soil extract broth**	**Electrical conductivity (dSm^−1^)**	**Media mineral composition (g L** ^**−1**^ **)**						
		**NaCl**	**MgCl**_2_ **. 6H**_2_ **O**	**MgSO**_4_ **. 7H**_2_ **O**	**KCl**	**NaHCO**_3_	**NaNO**_3_	**CaCl**_2_ **.2H**_2_ **O**
HSE-1	7.13	8.00	3.25	5.08	0.50	0.0250	0.100	0.08
HSE-2	9.73	8.88	3.61	5.64	0.555	0.0277	0.111	0.088
HSE-3	12.20	10.00	4.06	6.35	0.625	0.0310	0.125	0.100
HSE-4	16.59	11.40	4.64	7.25	0.71	0.0357	0.140	0.114
HSE-5	18.46	13.33	5.41	8.46	0.83	0.0416	0.166	0.133
HSE-6	21.29	16.00	6.50	10.16	1.00	0.050	0.200	0.16
HSE-7	24.8	20.00	8.125	12.7	1.25	0.0625	0.250	0.20
HSE-8	27.63	26.60	10.83	16.93	1.66	0.083	0.330	0.266
HSE-9	30.84	40.00	16.25	25.4	2.50	0.125	0.500	0.40
^1^HRA-control	119.8	80.00	32.5	52.8	5.00	0.250	1.00	0.80

^1^HRA, halophilic rhizospheric archaea medium.

#### 2.3.2. Growth, nutrient solubilization (p, k, and zn), and IAA production by a selected halophilic archaeon in different strength HSE broth

Based on the results of the experiment as described in Section 2.2, the best performing halophilic archaeon was selected for evaluating its growth, nutrient solubilization (P, K, and Zn) potential, and IAA production at different strengths of EC in HSE broth. The growth was quantified by estimating protein by Bradford's protein assay (Marion, 1976) as described above in Section 2.1. For quantifying IAA production and the solubilization of P, K, and Zn, the HSE broth was amended with tryptophan, tricalcium phosphate, potassium aluminum silicate, ZnO, and ZnCO_3_ similarly as described above in Section 2.1 for the modification of HRA medium. The quantification of IAA production and P, K, and Zn solubilization was carried out as per the method described in Section 2.1.

#### 2.3.3. Inoculum preparation for in planta studies

The inoculum for *in planta* studies was raised by culturing *H. pelagica* CDK2 in 250 ml of HRA1 broth for 15 days. The exponentially growing culture was centrifuged at 7,500 *g* for 15 min, and the supernatant was discarded. The pellet was washed twice with phosphate buffer saline (PBS) whose electrical conductivity was maintained at 8 dSm^−1^. The washed pellet was suspended in PBS (prepared using soil extract instead of water; EC: 8dsm^−1^) to maintain the cell density of ~10^9^ cells ml^−1^. The PBS with archaeal cells was mixed with charcoal (sterilized at 121°C for 20 min for 3 consecutive days and buffered with calcium carbonate) and homogenized. This mixture was air-dried overnight at room temperature under sterile conditions and was coated over wheat seeds immediately before sowing in the pots. For the soft mineral salt agar experiment, the seeds were soaked directly in the PBS with archaeal cell suspension for 30 min and were sown in the soft agar tubes.

#### 2.3.4. Soft agar experiment

Five wheat cultivars, namely two tolerant (K65 and KRL210) and three susceptible (HD2380, HD3086, and HD2687), were procured from ICAR-Indian Institute of Wheat and Barley Research, Karnal, India. A 21-day wheat-halophilic archaea interaction experiment was carried out in soft agar test tubes under controlled conditions to assess the effect of inoculating *H. pelagica* CDK2 on seed germination. Seeds of all the wheat cultivars were treated with cells of *H. pelagica* CDK2 suspended in PBS (halophilic archaeal soil extract phosphate buffer saline suspension; EC: 8 dsm^−1^) for 30 min. The mineral salt soil extract soft agar (MSSE) was prepared by dissolving various macro- and micronutrients, vitamins, and amino acids in previously prepared soil extract as per composition. The composition of MSSE was as follows: a) macronutrients (mg L^−1^): 1650.0 NH4NO3, 332.0 CaCl_2_, 180.690 MgSO_4_, 1900.0 KNO3, and 170.0 KH_2_PO_4_, b) micronutrients (mg L^−1^): 6.20 H_3_BO_3_, 0.025 CoCl_2_.6H_2_O, 0.025 CuSO_4_·5H_2_O, 37.30 disodium EDTA dihydrate, 27.800 FeSO_4_.7H_2_O, 16.90 MnSO_4_·H_2_O, 0.213 Na_2_MoO_4_·2H_2_O, 0.830 KI, 8.60, and ZnSO_4_.7H_2_O, c) vitamins (mg L^−1^): 100.0 myoinositol, 0.50 nicotinic acid, 0.50 pyridoxine HCl, and 0.10 thiamine HCl, and d) amino acid (mg L^−1^): 2.00 glycine and agar 8.0 gL^−1^ (pH 6.8 and EC: 8 dSm^−1^). The treated seeds were placed in a 50 ml test tube having 35 ml of MSSE soft agar medium (Filek et al., 2010). The tubes were incubated in the dark until seed germination and were later transferred to a glasshouse of Phytotron facility, IARI, New Delhi, under controlled conditions (glasshouse conditions were as follows; temperature: 20-24° C, humidity: 50%, and white LED lights, 15000 lux with 10 h of daily illumination). The un-inoculated control was maintained, and the experiment was carried out in three replications. Observations on vegetative growth were recorded after 21 days of incubation.

#### 2.3.5. Mesocosm study for plant–microbe interaction

The wheat–*H. pelagica* CDK2 interaction was further evaluated in a pot experiment to verify the effect of inoculating *H. pelagica* CDKA on wheat growth and yield parameters in an un-sterile rooting medium. The study was conducted in the net house of the Division of Microbiology, ICAR-Indian Agricultural Research Institute, New Delhi. The treatment included two salt-tolerant (K65 and KRL210) and three susceptible (HD2380, HD3086, and HD2687) wheat cultivars in triplicates. The seeds were coated with the halophilic archaeal-charcoal-based inoculum (as described in section 2.4) and were sown in 14-inch pots each containing 10 kg of un-sterile soil. The electrical conductivity of the soil was maintained at 8 dSm^−1^ using a mineral salt solution. An un-inoculated control was maintained for all the wheat cultivars in triplicates. The pots were fertilized with the recommended dose of NPK (120:60:60). The full dose of P, K, and 50% of N was applied as a basal dose before sowing. The remaining half dose of nitrogen was applied in 2 split doses at 21 d and 45 d. Observations on vegetative growth, osmolyte accumulation, and biochemical and yield parameters were recorded after 45 days of sowing and at the time of harvesting.

### 2.4. Estimation of vegetative and biochemical parameters

#### 2.4.1. Vegetative parameters

The shoot and root length (cm) and shoot and root biomass (g) were measured manually using standard laboratory protocols. The plants at 21 d from MSSE and at 45 d from pot experiments were sampled destructively. The roots were washed thoroughly with water to remove adhering medium and soil, respectively. The above-ground part (shoots) was separated from the roots and allowed to air dry for 1 h. The fresh weight (biomass) and the length of the shoot and roots were measured manually with a balance and sliding caliper, respectively.

#### 2.4.2. Quantification of crude protein

Crude protein was extracted from 100 mg wheat leaves homogenized with the 5 ml salt-alkaline phosphate extraction buffer (0.1 M NaOH in 3.5% NaCl; pH 7.4). The homogenate was incubated at 60 °C for 90 min followed by centrifugation at 4,000 *g* for 20 min at room temperature. A total of 1 ml of supernatant was used for the quantification of crude protein following Bradford's quantitative protein extraction method (Marion, 1976). The protein content was expressed as μg protein mg^−1^ of wheat tissue. The quantity of protein in the sample was extrapolated from a standard curve prepared using different concentrations of bovine serum albumin (BSA).

#### 2.4.3. Quantification of total chlorophyll

Chlorophyll from the leaf sample was estimated using the DMSO method (Blanke, 1992). A 100 mg fresh plant leaf sample was placed in a 100 ml volumetric flask containing 10 ml DMSO (Dimethyl sulfoxide). The flask was allowed to stand overnight before measuring the spectroscopic absorbance at 663 and 645 nm. DMSO without leaf sample was used as blank, and un-inoculated wheat leaf was used as control. The total chlorophyll content of the wheat leaf was calculated using the formula given below.


$$\begin{array}{c} mg\;of\;chlorophyll\;g^{-1}\;leaf\;tissue\;=20.2\left(A645\right)+\\ 8.02{\left(A663\right)}^{*}\frac{volume\;of\;DMSO}{1000^{*}weight\;of\;leaf\;sample} \end{array}$$

#### 2.4.4. Quantification of total sugars and proline accumulation

Total sugars in wheat leaf samples were quantified using the phenol-sulfuric acid method (Dubois et al., 1951). D-glucose was used as a standard for the preparation of the standard curve, and a run without a sample was used as a blank. The amount of proline synthesized in wheat seedlings in response to salinity stress was estimated using an acid ninhydrin-sulfosalicylic acid method as described by Ábrahám and László Erdei (2010). A blank was run without the sample, and a standard graph was prepared using L-proline.

#### 2.4.5. Quantification of antioxidant enzyme activity

The antioxidant enzymes, such as ascorbate peroxidase (APox), catalase (CAT), and superoxide dismutase (SOD) accumulation, were quantified in wheat leaves. The enzyme extract was prepared by freezing 1 g of wheat leaf sample in liquid nitrogen to arrest the proteolytic activity followed by grinding with 10 ml of extraction buffer (0.1 M phosphate buffer, pH 7.5, containing 0.5 mM EDTA with 1 mM ascorbic acid). The mixture was passed through four-layered muslin cloth followed by centrifugation at 15,000 *g* for 20 min. The resultant filtrate was used as an enzyme in the enzyme assay. The APox activity was determined by measuring the decrease in ascorbic acid content as described by Nakano and Asada (1981). Superoxide dismutase activity was quantified by measuring the decrease in the absorbance of formazan generated by superoxide radicles and nitroblue tetrazolium (NBT) dye as per the method described by Dhindsa et al. (1981). A unit of SOD activity was expressed as the amount of enzyme, which reduced the absorbance to 50% as compared to tubes lacking enzyme. Catalase antioxidant enzyme activity was analyzed by calculating the amount of H_2_O_2_ decomposed by comparing it to a standard curve of known concentrations of hydrogen peroxide (Aebi, 1984).

#### 2.4.6. Estimation of root architecture parameters at harvesting

Analysis of changes in root physiological parameters at the harvesting was performed using a root scanner LA2400 Instruments REGENT, equipped with WinRhizo software. Freshly harvested roots were washed under running tap water to remove all the soil particles attached to the roots. After washing, the roots were kept immersed in water in a 500 ml beaker and taken for root imaging. Roots were placed horizontally on the scanner block filled with a minimal amount of distilled water to keep the roots submerged. The entangling of roots was removed using the forceps.

### 2.5. Quantification of yield parameters at harvest

To quantify yield parameters, such as grain yield per plant and grains per spike, the plants were harvested after grain filling and drying. The spikes were manually separated from the harvested plants, and the data for grain content per spike were recorded. The grains separated from spikes of a single plant were combined to extrapolate grain yield per plant manually.

### 2.6. Statistical analysis of data

All the experiments were carried out in triplicates, and statistical analysis was performed using WASP 2.0 (Web Agri Stat Package, Indian Council of Agricultural Research, India). The data were subjected to ANOVA, and the least significant differences (LSDs) at *P* ≤ 0.05 and *P* ≤ 0.01 for *in planta* and *in vitro* studies, respectively, among means were compared using standard deviation.

## 3. Results

### 3.1. Growth of halophilic archaea in halophilic broth

All 28 halophilic archaeal isolates exhibited growth in the HRA broth. The production of protein after 10 days varied significantly among these isolates (Supplementary Table 1). The protein production in the halophilic broth ranged from 122.371 μg ml^−1^ to 271.80 μg ml^−1^. The highest protein production was observed in *Halococcus hamelinensis* IARI-SNS2 (271.8 μg ml^−1^), followed by *Halococcus* sp. IARI-BGAK2 (268.94 μg ml^−1^), *Natrialba* sp. IARI-SGAB2 (257.51 μg ml^−1^), *Halolamina pelagica* CDK2 (252.65 ± 8.33 μg ml^−1^), *Haloferax volcanii* IARI-CFAB4 (254.08 ± 1.81 μg ml^−1^), and *Halogeometricum borinquense* IARI-WRAK9 (255.22 ± 7.96 μg ml^−1^). The protein production of *H. pelagica* IARI-CDK2, *H. volcanii* IARI-CFAB4, and *H. borinquense* IARI-WRAK9 did not significantly differ from each other (252.65, 253.08, and 255.22 μg ml^−1^, respectively). The lowest growth in terms of protein production was observed in *Natrinema altunense* IARI-WRAK5 (122.37 μg ml^−1^).

### 3.2. Quantification of plant growth-promoting properties of halophilic archaea

#### 3.2.1. Qualitative estimation of P, K, and Zn solubilization

Based on the halo zone formation observed around spot-inoculated colonies in specific solubilization media (HPS, HKS, and HZS medium), we qualitatively assessed the ability of 28 halophilic archaea to solubilize phosphorus (P), potassium (K), and zinc (Zn) (Supplementary Table 2). Among the isolates, 17 showed significant zones of P solubilization. The highest D/D ratio (cm) for P solubilization was observed in *H. pelagica* CDK2 (1.8), followed by *Halobacterium* sp. IARI-SNS3 (1.75), *Natronoarchaeum mannanilyticum* IARI-SSAB3 (1.6), *H. borinquense* IARI-WRAK9 (1.6), *Halolamina pelagica* IARI-CSK1 (1.6), and *Natronomonas pharaonis* IARI-MAAB4 (1.6). The halophilic archaea *N. mannanilyticum* IARI-SSAB3, *H. borinquense* IARI-WRAK9, *H. pelagica* IARI-CSK1, and *N. pharaonis* IARI-MAAB4 exhibited similar D/D ratios for P solubilization. Conversely, the lowest D/D ratio was observed in *Halorubrum* sp. IARI-WRAB4 (1.16), followed by *Halostagnicola kamekurae* IARI-TWAK7 (1.2) and *Halosarcina* sp. IARI-WRAB3 (1.25), respectively. Furthermore, 21 isolates displayed significant K solubilization zones, with D/D ratios ranging from 1.25 to 1.68. The highest D/D ratios for K solubilization were recorded in *Haloterrigena* sp. IARI-SOAB2, *N. pharaonis* IARI-MAAB4, and *H. pelagica* CDK2 (1.68, 1.66, and 1.65, respectively). In contrast, only a few isolates demonstrated the ability to solubilize Zn, with six isolates solubilizing ZnO and three isolates solubilizing ZnCO3 in the respective HZS medium. The D/D ratio for all the Zn solubilizing isolates was just above 1, indicating the formation of very small clearing zones around the spot-inoculated colonies.

#### 3.2.2. Quantitative estimation of P, K, and Zn solubilization and effect on medium ph

**Figure 1:**
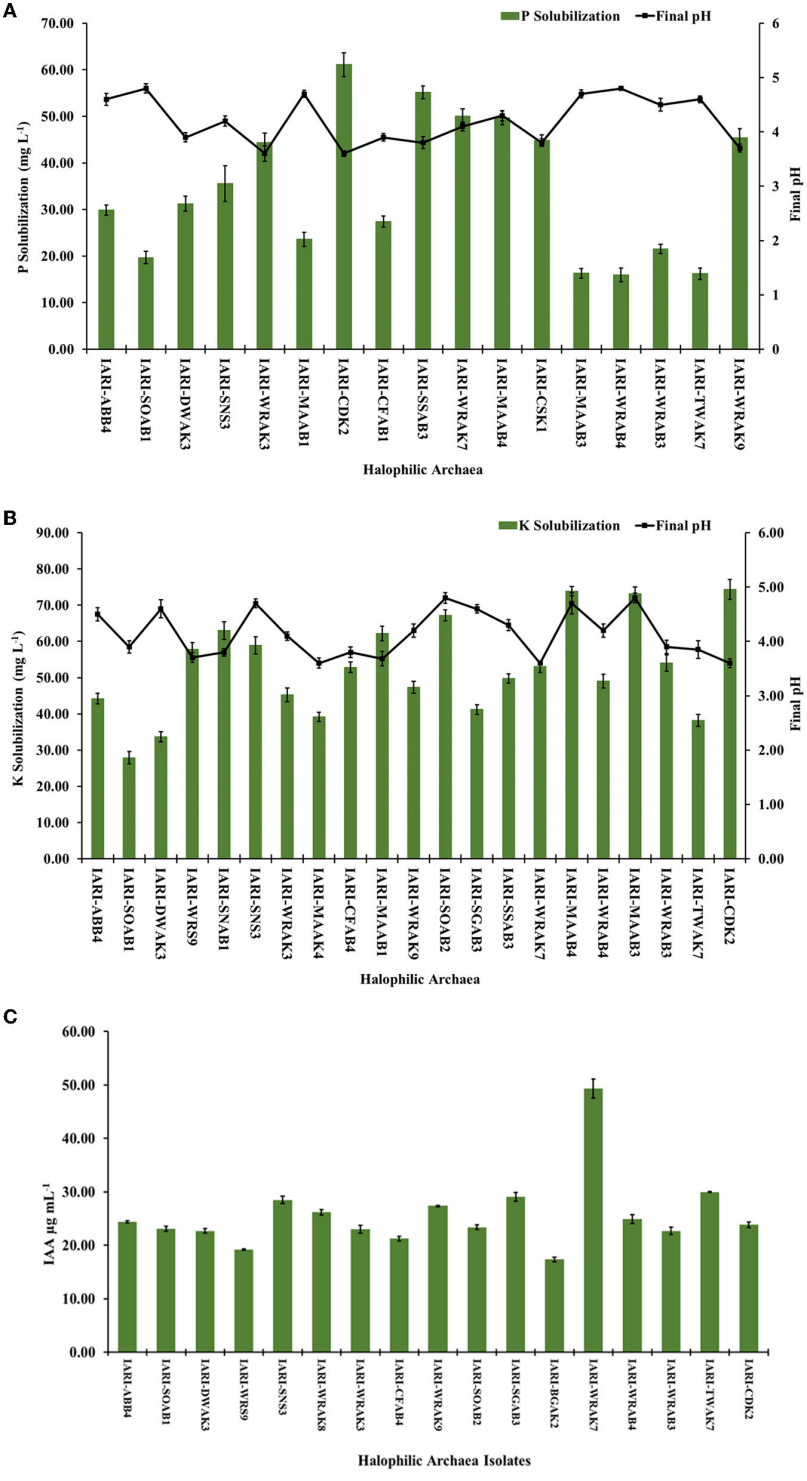
Quantification of **(A)** P and **(B)** K solubilization and reduction in pH of the respective modified HRA media by selected isolates of halophilic archaea after 10 d of growth (LSD *p* ≤ 0.05: for P and K solubilization between isolates is 2.98 and 2.544, respectively; for pH reduction: for P and K solubilization between isolates is 0.133 and 0.165, respectively); **(C)** quantification of indole acetic acid production by the selected halophilic archaea isolates grown in HRA broth supplemented with L-tryptophane (LSD *p* ≤ 0.05: 1.46; error bar denotes standard deviation from the mean).

We conducted quantification of P solubilization and measured the reduction in pH of the medium for the selected 17 isolates, grown in HPS broth. On the 10th day of growth, a significant decrease in pH was observed, ranging from 3.6 to 4.8, compared to the initial pH of 7.2. The greatest reduction in pH was observed in *Haloarcula tradensis* IARI-WRAK3 and *H. pelagica* CDK2 (pH: 3.6), while the least reduction occurred in *Halorubrum* sp. IARI-WRAB4 and *Haloarcula argentinensis* IARI-SOAB1 (pH: 4.8) (Figure 1A). Regarding P solubilization in HPS broth, the values ranged from 15.98 ± 1.46 to 61.10 ± 2.56 mg L^−1^ among the 17 isolates. *H. pelagica* IARI-CDK2 demonstrated the highest P solubilization, significantly measuring 61.10 ± 2.56 mg L^−1^. Close behind were *N. mannanilyticum* IARI-SSAB3 with 55.17 ± 1.36 mg L^−1^, *Halogeometricum rufum* IARI-WRAK7 with 50.04 ± 1.56 mg L^−1^, and *N. pharaonis* IARI-MAAB4 with 49.69 ± 1.45 mg L^−1^ (as depicted in Figure 1A). Conversely, the lowest P solubilization was observed in *Halorubrum* sp. IARI-WRAB4 (15.98 ± 1.46 mg L^−1^), *H. kamekurae* IARI-TWAK7 (16.20 ± 1.21 mg L^−1^), *Halopenitus persicus* IARI-MAAB3 (16.30 ± 1.06 mg L^−1^), and *H. argentinensis* IARI-SOAB1 (19.68 ± 1.35 mg L^−1^) (Figure 1A).

Similarly, a significant reduction in the pH of HKS broth was observed on the 10th day of incubation, compared to the initial pH of 7.4, during the solubilization of P. The highest pH reduction was observed in *Haloferax* sp. IARI-MAAK4, *H. rufum* IARI-WRAK7, and *H. pelagica* CDK2 (pH: 3.6), followed by *Haloferax alexandrinus* IARI-MAAB1 (pH: 3.68) and *Natrinema* sp. IARI-WRS9 (pH: 3.7) (as depicted in Figure 1B). Conversely, the least decrease in pH was observed in *Halopenitus persicus* IARI-MAAB3 and *Haloterrigena hispanica* IARI-SGAB3 (pH: 4.8), followed by *Halobacterium* sp. IARI-SNS3 and *N. pharaonis* IARI-MAAB4 (pH: 4.7) (refer to Figure 1B). For potassium solubilization in HKS broth, the halophilic archaea exhibited a range of solubilization from 27.89 ± 0.30 to 74.36 ± 2.74 mg L^−1^. *H. pelagica* CDK2 (74.36 ± 2.74 mg L^−1^), *N. pharaonis* IARI-MAAB4 (73.85 ± 1.26 mg L^−1^), and *H. persicus* IARI-MAAB3 (73.16 ± 1.84 mg L^−1^) solubilized a significantly high amount of K. On the other hand, *H. argentinensis* IARI-SOAB1 (27.89 ± 0.30 mg L^−1^), *Haloarcula* sp. IARI-DWAK3 (33.64 ± 0.39 mg L^−1^), and *H. kamekurae* IARI-TWAK7 (38.19 ± 0.06 mg L^−1^) solubilized a significantly low amount of K (Figure 1B).

The solubilization of Zn by six isolates in the HZS medium using ZnO as the source ranged from 4.96 ± 0.03 mg L^−1^ to 7.21 ± 0.29 mg L^−1^. Additionally, in the HZS medium with ZnCO3 as the source, three positive isolates exhibited Zn solubilization ranging from 4.91 ± 0.15 to 5.84 ± 0.25 mg L^−1^. Notably, *H. borinquense* IARI-WRAK9, *H. pelagica* CDK2, and *Natrinema pallidum* IARI-WRAK8 were capable of solubilizing both insoluble sources of Zn (ZnO and ZnCO3), whereas *H. alexandrinus* IARI-MAAB1, *N. pharaonis* IARI-MAAB4, and *Halorubrum* sp. IARI-WRAB4 solubilized only ZnO. Among the ZnO-containing HZS media, *H. pelagica* CDK2 exhibited the maximum quantity of Zn solubilization (7.21 ± 0.29 mg L^−1^), which was statistically comparable to *Halorubrum* sp. IARI-WRAB4 (7.14 ± 0.21 mg L^−1^) and significantly higher than IARI-MAAB4 (5.72 ± 0.09 mg L^−1^), IARI-MAAB1 (5.28 ± 0.98 mg L^−1^), IARI-WRAK8 (5.4 ± 0.20 mg L^−1^), and IARI-WRAK9 (4.96 ± 0.23 mg L^−1^). In contrast, *N. pallidum* IARI-WRAK8 exhibited the highest solubilization of Zn in the HZS media containing ZnCO3 (5.84 ± 0.25 mg L^−1^), which was significantly higher than the solubilization by *H. pelagica* IARI-CDK2 (5.27 ± 0.12 mg L^−1^) and IARI-WRAK9 (4.91 ± 0.15 mg L^−1^). A significant reduction in pH was observed in the HRA medium containing ZnO and ZnCO3 compared to the initial pH of 7.4, ranging from 3.6 ± 0.15 to 4.2 ± 0.13.

#### 3.2.3. Phytohormone production (IAA production)

The ability of all halophilic archaea isolates to produce the phytohormone IAA (indole-3-acetic acid) was assessed by culturing them in HRA media supplemented with tryptophan. Out of the isolates, 17 exhibited significant production of IAA, ranging from 17.33 ± 0.43 μg ml^−1^ to 49.31 ± 1.79 μg ml^−1^ (Figure 1C). Among them, the highest amount of IAA was produced by *H. rufum* IARI-WRAK7 (49.31 ± 1.79 μg ml^−1^), which was significantly greater than the IAA production of all other isolates. Following closely were *H. kamekurae* IARI-TWAK7 (29.97 ± 0.79 μg ml^−1^), *H. hispanica* IARI-SGAB3 (29.06 ± 0.82 μg ml^−1^), and IARI-SNS3 (28.49 μg ml^−1^). The isolate *Halolamina pelagica* CDK2, which exhibited significant solubilization of P, K, and Zn, also produced IAA (23.86 ± 0.52 μg ml^−1^). On the other hand, *Halococcus* sp. IARI-BGAK2 produced the lowest amount of IAA, which was significantly measured at 17.33 ± 0.43 μg ml^−1^.

#### 3.2.4. Quantification of growth and plant growth-promoting properties of a selected halophilic archaeon in halophilic soil extract broth having different salinity concentrations

**Figure 2:**
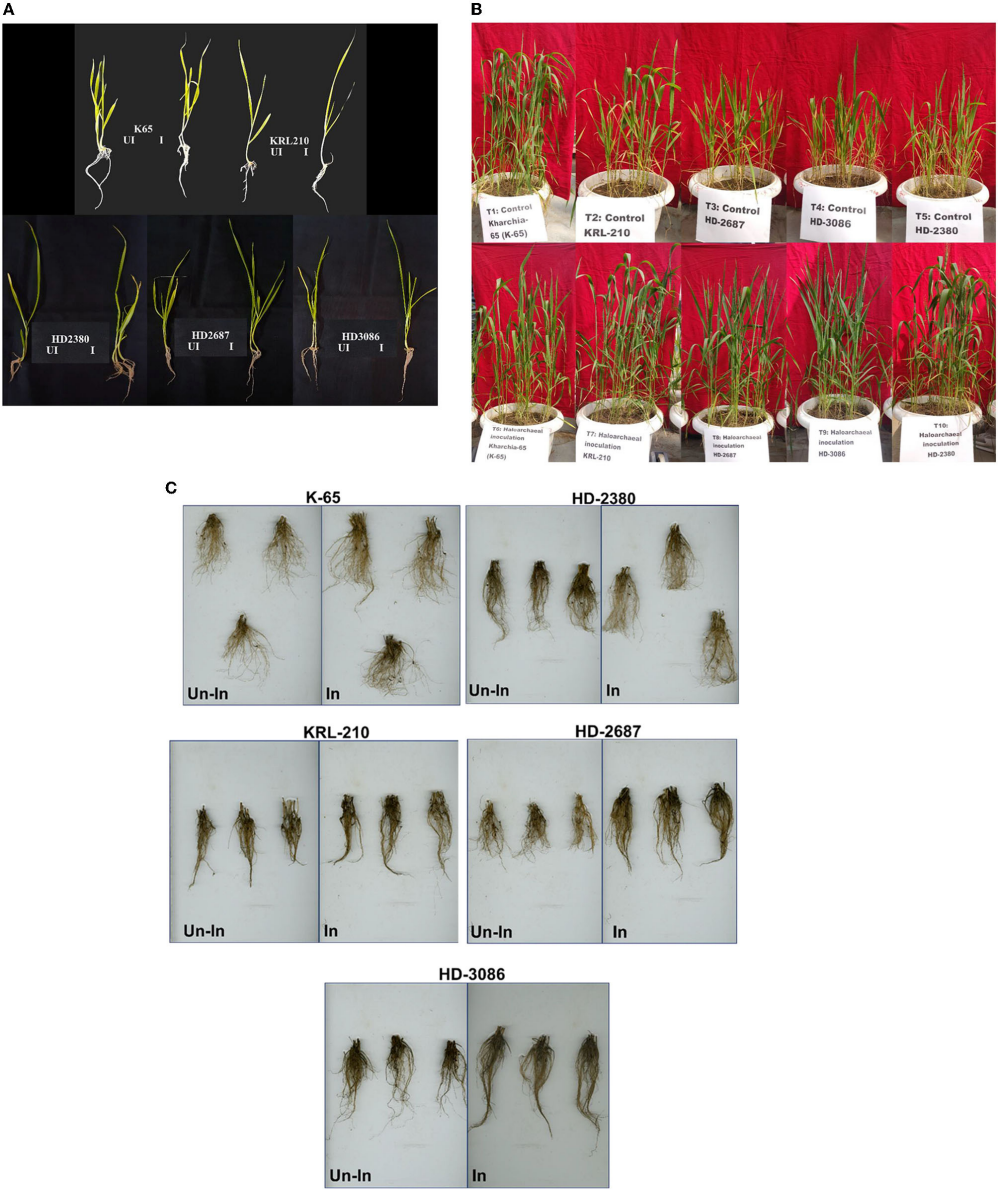
Wheat seedlings growth as influenced by inoculation of *Halolamina pelagica* CDK2 **(A)** at 21st day of seed germination in soft agar having EC of 8 dS m-1, **(B)** effect of inoculating *Halolamina pelagica* CDK2 on the growth of different wheat cultivars at 45th d of seed germination, and **(C)** effect of inoculating *Halolamina pelagica* CDK2 on root architecture of different wheat cultivars at harvesting (Un-In: un-inoculated; In: inoculated).

Based on the obtained results, *H. pelagica* CDK2 was chosen for further investigation. The study aimed to assess its ability to thrive in lower electrical conductivity (EC) conditions, compared to its natural habitat with EC ranging from 75.25 to 106.7 dSm^−1^. Additionally, the study aimed to evaluate its potential in enhancing wheat growth by managing nutrient availability and mitigating salinity stress. A modified growth medium suitable for halophilic organisms, supplemented with soil extract (HSE), was utilized. The HSE medium was prepared with different levels of EC, as shown in Table 1. The growth and plant growth-promoting (PGP) attributes of *H. pelagica* CDK2 were quantified at 8-, 15-, and 21-day intervals, as outlined in Table 2. Observations revealed that *H. pelagica* CDK2 exhibited growth even at the lowest EC of 7.13 dSm^−1^. Protein production by the haloarchaeon increased significantly as the electrical conductivity (EC) of the HSE broth increased (Table 2). Notably, at the highest EC level of 119.8 dSm^−1^, *H. pelagica* CDK2 produced the highest amount of protein throughout the incubation period. Specifically, protein production reached 220.80 ± 2.35 μg ml^−1^, 243.25 ± 10.40 μg ml^−1^, and 263.45 ± 10.10 μg ml^−1^ at 8, 15, and 21 days, respectively (Table 2 and Figure 2A).

**Table 2:** Quantification of growth (protein mg ml^−1^), nutrient solubilization (P, K, and Zn), and indole acetic acid production by *Halolamina pelagica* CDK2 in different strength halophilic soil extract (HSE) broth.

**HSE Broth**	**Electrical conductivity (dSm^−1^)**	**Growth (protein mg ml** ^**−1**^ **)**			**Nutrient solubilization (mg L** ^**−1**^ **)** ^*^				IAA^*^production (μg ml^−1^)
		**8 d**	**15 d**	**21 d**	**P**	**K**	**ZnO**	**ZnCO**_3_	
HSE-1	7.13	15.22 ± 0.02	28.25 ± 0.89	46.11 ± 0.86	8.12 ± 0.20	ND	ND	ND	2.64 ± 0.03
HSE-2	9.73	29.01 ± 0.52	32.14 ± 0.90	56.14 ± 0.48	9.24 ± 0.30	3.67 ± 0.04	ND	ND	3.63 ± 0.11
HSE-3	12.20	43.70 ± 0.59	51.97 ± 0.69	86.17 ± 0.22	10.48 ± 0.60	4.92 ± 0.12	ND	ND	4.92 ± 0.15
HSE-4	16.59	52.85 ± 0.54	66.91 ± 0.52	97.71 ± 1.97	12.89 ± 0.30	8.41 ± 0.37	1.25 ± 0.02	ND	5.89 ± 0.12
HSE-5	18.46	51.60 ± 1.63	80.77 ± 0.49	103.11 ± 4.26	14.76 ± 0.27	12.49 ± 0.42	1.7 ± 0.03	ND	7.01 ± 0.032
HSE-6	21.29	59.85 ± 1.34	86.68 ± 3.18	110.65 ± 2.92	17.16 ± 0.41	18.38 ± 0.58	1.93 ± 0.03	ND	8.02 ± 0.11
HSE-7	24.8	70.69 ± 2.14	103.45 ± 2.45	122.48 ± 0.10	22.63 ± 0.70	21.35 ± 0.20	2.31 ± 0.04	ND	11.63 ± 0.35
HSE-8	27.63	106.77 ± 0.65	143.25 ± 5.57	171.08 ± 6.22	27.71 ± 0.88	27.11 ± 0.41	2.96 ± 0.05	ND	15.42 ± 0.40
HSE-9	30.84	193.31 ± 5.32	209.34 ± 7.45	246.48 ± 8.35	31.44 ± 1.17	42.09 ± 1.44	3.54 ± 0.10	2.47 ± 0.02	16.98 ± 0.20
^**^HRA-control	119.8	220.80 ± 2.35	243.25 ± 10.40	263.45 ± 10.10	61.10 ± 2.56	74.36 ± 2.74	7.21 ± 0.29	5.27 ± 0.12	23.85 ± 0.52
**LSD** ***p** **≤*** **0.01**		Factor A (EC Level): 5.206; Factor B (Days of incubation: 2.859; Interaction (AxB): 9.027	1.23	1.78	2.17	0.012	0.55

^*^The nutrient solubilization (P, K, and Zn mg L^−1^) and IAA μg ml^−1^ were quantified after 10 d of incubation. ^*^HRA, halophilic rhizospheric archaea medium.

*H. pelagica* CDK2 exhibited the ability to solubilize insoluble tricalcium phosphate across various salinity levels, ranging from EC: 7.13 to 30.84 dSm^−1^ (HSE-1 to HSE-9). The solubilization of phosphorus (P) in different EC levels of HSE broth ranged from 8.12 ± 0.19 mg L^−1^ (EC: 7.13 dSm^−1^) to 31.44 ± 1.17 mg L^−1^ (EC: 30.84 dSm^−1^) after 10 days. The lowest values of P solubilization in HSE-1 broth (EC: 7.13 dSm^−1^) were statistically similar to those observed in HSE-2, with a P solubilization of 8.12 ± 0.20 mg L^−1^ at an EC of 9.73 dSm^−1^. However, a significant increase in P solubilization was observed as the EC level increased from 12.20 to 30.84 dSm^−1^. The highest solubilization, reaching 31.44 ± 1.17 mg L^−1^, was observed in HSE-9 with an EC of 30.84 dSm^−1^. It is worth noting that the P solubilization in HSE-9 was significantly lower compared to the solubilization observed in the control HRA media (61.10 ± 2.56 mg L^−1^) with an EC of 119.8 dSm^−1^ (Table 2). A similar pattern was observed for potassium (K) solubilization, with the exception that *H. pelagica* CDK2 did not exhibit K solubilization in the HSE-1 medium with the lowest EC of 7.13 dSm^−1^. However, K solubilization began at a level of 3.67 ± 0.036 mg L^−1^ in HSE-2 with an EC of 9.73 dSm^−1^, and this was statistically similar to the K solubilization (4.92 ± 0.12 mg L^−1^) observed in HSE-3 with an EC of 12.20 dSm^−1^. A significant increase in K solubilization was observed as the EC increased from 16.59 to 30.84 dSm^−1^, reaching a maximum yield of 42.09 ± 1.44 mg L^−1^ in HSE-9 medium with an EC of 30.84 dSm^−1^. However, this value was significantly lower than the K solubilization observed in the control HRA medium (74.36 ± 2.74 mg L^−1^) with an EC of 119.8 dSm^−1^ (Table 2).

In contrast to P and K solubilization, the solubilization of zinc (Zn) by *H. pelagica* CDK2 in HSE media using ZnO as the source of Zn was not observed at lower EC levels of 7.13, 9.73, and 12.20 dSm^−1^ (HSE broth 1, 2, and 3, respectively). Solubilization of ZnO was statistically similar (ranging from 1.25 ± 0.02 to 2.96 ± 0.05 mg L^−1^) in media with ECs ranging from 16.59 to 27.63 dSm^−1^ (HSE-4 to HSE-8). The maximum solubilization of zinc oxide (ZnO) was observed in the medium with an EC of 30.84 dSm^−1^, specifically in the HSE-9 medium, with a solubilization level of 3.54 ± 0.10 mg L^−1^. However, this value was significantly lower than the solubilization observed in the control HRA medium with an EC of 119.8 dSm^−1^. Furthermore, *H. pelagica* CDK2 did not exhibit solubilization of zinc carbonate (ZnCO3) at any of the lower EC levels. It only showed solubilization in the medium with an EC of 30.84 dSm^−1^, with a solubilization level of 2.47 ± 0.015 mg L^−1^. Again, this value was significantly lower than the solubilization observed in the control HRA medium with an EC of 119.8 dSm^−1^ (Table 2).

The production of indole acetic acid (IAA) was observed at all EC levels in different HSE broths. There was a significant increase in indole-3-acetic acid (IAA) production with each subsequent increase in the electrical conductivity (EC) level, as indicated in Table 2. The IAA production in HSE broth supplemented with tryptophan ranged from 2.64 ± 0.027 μg ml^−1^ in HSE-1 medium with an EC of 7.13 dSm^−1^ to 16.98 ± 0.20 μg ml^−1^ in HSE-9 medium with an EC of 30.84 dSm^−1^ on the 10th day of incubation. Notably, the IAA production in the control HRA medium (23.85 ± 0.52 μg ml^−1^) was significantly higher compared to the values observed in the HSE-9 medium (16.98 ± 0.20 μg ml^−1^) (Table 2).

#### 3.2.5. Effect of *H. pelagica* CDK2 inoculation on germination parameters of different wheat cultivars in MS-soft agar

Inoculation of the halophilic archaea *H. pelagica* CDK2 had a significant positive impact on the germination and initial vegetative growth of all wheat cultivars grown in soft agar (EC 8 dSm^−1^) compared to the un-inoculated control (Figure 2A). The germination time of the inoculated wheat cultivars was significantly reduced by 2 to 3 days compared to the germination observed on the 8th day in the un-inoculated control treatment. Among the inoculated treatments, wheat cultivar K65 exhibited the fastest germination on the 5th day, followed by germination on the 6th day for all other cultivars. On the 21st day of germination, shoot and root growth parameters were measured (Supplementary Table 3). Inoculation resulted in a significant increase in the total shoot weight and length of all wheat cultivars compared to their respective un-inoculated treatments. The maximum percent increase in shoot weight due to inoculation was observed in wheat cultivar HD2687 (21.23%), followed by HD2380 (18.48%), K65 (16.96%), HD3086 (15.93%), and KRL210 (13.78%) compared to their respective un-inoculated wheat cultivars. Similarly, the maximum percent increase in shoot length due to inoculation was observed in wheat cultivar K65 (25.19%), followed by HD3086 (21.45%), HD2687 (18.80%), HD2380 (17.82%), and KRL210 (15.24%) compared to their respective un-inoculated wheat cultivars.

Likewise, inoculation of *H. pelagica* CDK2 in different wheat cultivars significantly improved the root growth parameters compared to their respective un-inoculated wheat cultivars. Inoculation significantly increased the percent root weight of wheat cultivar K65 by 67.18%, followed by HD3086 (47.06%), HD2687 (46.56%), KRL210 (42.30%), and HD2380 (42.22%) compared to their respective un-inoculated wheat cultivars. The number of lateral roots also increased compared to the un-inoculated control (Figure 2A). A similar effect was observed on total root length, and inoculation of *H. pelagica* CDK2 significantly increased the percent root length of wheat cultivar HD2380 by 39.93%, followed by HD2687 (37.81%), HD3086 (33.53%), K65 (16.45%), and KRL210 (14.62%) compared to their respective un-inoculated wheat cultivars.

### 3.3. Pot evaluation of the effect of inoculating *H. pelagica* CDK2 in different wheat cultivars

#### 3.3.1. Shoot and root parameters on the 45th day of seed germination

Inoculation with *H. pelagica* CDK2 significantly increased the fresh shoot biomass and shoot length of all wheat cultivars compared to their respective un-inoculated treatments on the 45th day after seed germination (Table 3). The wheat cultivar HD3086 showed the maximum percent increase in shoot biomass due to inoculation (47.13%), followed by HD2380 (32.44%), KRL210 (32.37%), HD2687 (30.65%), and K65 (28.81%) compared to their respective un-inoculated cultivars. Similarly, the maximum percent increase in shoot length due to inoculation was observed in wheat cultivar KRL210 (10.32%), followed by HD3086 (9.30%), K65 (6.32%), HD2380 (4.94%), and HD2687 (4.25%) over their respective un-inoculated treatments (Table 3). The differences in shoot biomass and shoot height in response to halophilic archaeal inoculation were statistically significant among the wheat cultivars at *p* ≤ 0.05. A representative image of the pot experimental study is shown in Figure 2B.

**Table 3:** Quantification of vegetative growth and yield parameters in different wheat cultivars (pot experiment) as influenced by inoculation of *H. pelagica* CDK2.

**Cultivars**	**Root biomass (g)**		**Shoot biomass (g)**		**Root length (cm)**		**Shoot height (cm)**		**Grains per spike**		**Grain yield (g/plant)**	
	**UI** ^**^	**I**	**UI**	**I**	**UI**	**I**	**UI**	**I**	**UI**	**I**	**UI**	**I**
K65	3.09 ± 0.57	4.13 ± 0.22	32.29 ± 0.83	41.6 ± 1.46	21.86 ± 0.37	25.06 ± 0.46	69.63 ± 0.57	74.03 ± 0.21	49.67 ± 0.57	55.33 ± 1.52	3.47 ± 0.01	5.21 ± 0.04
KRL210	3.26 ± 0.38	4.53 ± 0.27	36.24 ± 0.67	47.97 ± 1.79	22.23 ± 0.10	25.16 ± 0.35	67.46 ± 0.76	74.43 ± 0.49	52.00 ± 1.01	56.00 ± 2.64	3.33 ± 0.12	4.92 ± 0.14
HD2380	1.89 ± 0.35	2.44 ± 0.45	26.20 ± 0.35	34.70 ± 2.02	22.55 ± 0.13	25.06 ± 0.47	66.73 ± 0.40	70.03 ± 0.28	45.33 ± 1.69	51.67 ± 1.15	2.83 ± 0.05	3.68 ± 0.05
HD3086	2.65 ± 0.42	4.03 ± 0.5	25.25 ± 0.59	37.15 ± 1.81	19.46 ± 0.11	27.48 ± 1.24	60.2 ± 0.96	65.8 ± 0.4	44.00 ± 1.0	52.67 ± 2.08	3.31 ± 0.08	4.59 ± 0.06
HD2687	2.09 ± 0.43	3.06 ± 0.35	24.83 ± 1.31	32.44 ± 1.04	15.03 ± 0.66	19.36 ± 0.92	59.5 ± 1.13	62.03 ± 0.15	48.33 ± 0.58	58.00 ± 2.01	2.70 ± 0.03	3.83 ± 0.05
LSD*p ≤* 0.05												
Cultivars A	0.487		1.675		0.723		0.788		1.992		0.084	
Treatment B	0.308		1.057		0.467		0.493		1.261		0.054	
Interaction AxB	0.683		2.366		1.03		1.108		2.825		0.114	

^**^UI, un-inoculated control; I, inoculated treatment.

In a similar manner to the shoot parameters, inoculation of *H. pelagica* CDK2 significantly improved root growth in different wheat cultivars compared to their respective un-inoculated treatments (Table 3). The maximum significant increase in root biomass (%) due to inoculation was observed in wheat cultivar HD3086 (52.08%), followed by HD2687 (46.41%), KRL210 (38.96%), K65 (33.66%), and HD2380 (29.10%) compared to their respective un-inoculated treatments (Table 3). The percent increase in root biomass in inoculated HD3086 was significantly higher than the percent increase observed in other wheat cultivars. Similarly, inoculation significantly increased the root length of all wheat cultivars compared to the un-inoculated treatments. The maximum root length due to inoculation was observed in wheat cultivar HD3086 (27.48 cm). Among the inoculated cultivars, KRL210, K65, and HD2380 showed statistically similar root lengths (25.16 cm, 25.06 cm, and 25.06 cm, respectively). The least increase in root length was observed in cultivar HD2687 (19.36 cm). The percent increase in root length due to inoculation was maximum in HD3086 (41.21%), followed by HD2687 (28.81%), K65 (14.63%), KRL210 (13.18%), and HD2380 (11.13%) compared to their respective un-inoculated wheat cultivars (Table 3).

#### 3.3.2. Root architecture at harvest time

**Figure 3:**
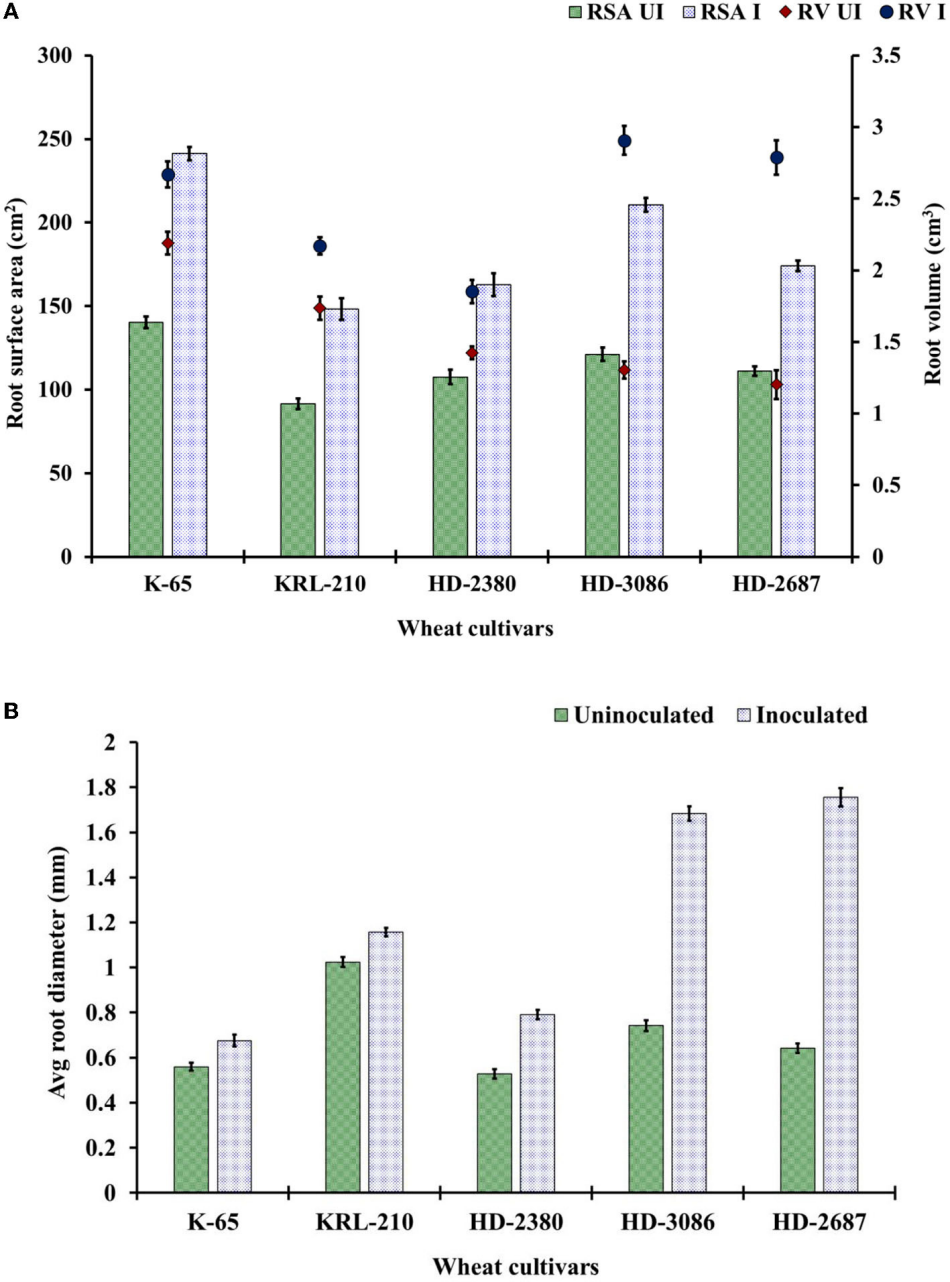
Root parameters of wheat cultivars: **(A)** root surface area (RSA) (cm^2^) and the total root volume (RV) (cm^3^) and **(B)** average root diameter as influenced by the seed inoculation of *H. pelagica* CDK2 in different wheat cultivars (UI: un-inoculated; I: inoculated). (LSD *p* ≤ 0.05 for root surface area: Factor A (Cultivars): 5.20, Factor B (Treatments): 3.29, Interaction AxB: 7.36; LSD *p* ≤ 0.05 for root volume: Factor A (Cultivars): 0.077, Factor B (Treatments): 0.042, Interaction AxB: 0.107, LSD *p* ≤ 0.05 for average root diameter: Factor A (Cultivars): 0.036, Factor B (Treatments): 0.029, Interaction AxB: 0.04; error bar represents standard deviation).

The root scanning revealed significant increases in the number of lateral roots, total root surface area, and root volume due to the inoculation of *H. pelagica* CDK2 compared to the un-inoculated control at harvest time (Figure 3). The root surface area in the inoculated wheat treatments at harvest time ranged from 148.34 ± 6.41 cm^2^ to 241.25 ± 3.91 cm^2^, which was significantly higher than their respective un-inoculated control treatments (ranging from 91.67 ± 1.15 cm^2^ to 140 ± 2.88 cm^2^). The overall percent increase in root surface area ranged from 51.34% to 73.75%. The wheat cultivar HD3086 showed the maximum percent increase in root surface area (73.75%), followed by K65 (71.96%), KRL210 (61.82%), HD2687 (56.42%), and HD2380 (51.34%) compared to their respective un-inoculated control (Figure 3A).

The total root volume of the different wheat cultivars significantly increased compared to their respective un-inoculated cultivars. The root volume in the treated wheat cultivars at harvest time ranged from 1.85 ± 0.061 cm^3^ to 2.91 ± 0.12 cm^3^, which was significantly higher than their respective control treatments (ranging from 1.20 ± 0.018 cm^3^ to 2.19 ± 0.080 cm^3^) (Figure 3A). The overall percent increase in root volume ranged from 21.97% to 131.83%. The maximum percent increase in root volume due to inoculation was observed in wheat cultivar HD2687 (131.83%), followed by HD3086 (71.96%). Cultivar HD2380, KRL210, and K65 showed significantly less percent increase due to inoculation (30.14%, 25.11%, and 21.97%, respectively) compared to HD2687 and HD3086. The average root diameter in each of the *H. pelagica* CDK2-treated wheat cultivars (ranging from 0.68 ± 0.026 mm to 1.76 ± 0.025 mm) increased significantly compared to their respective non-treated wheat cultivars (ranging from 0.53 ± 0.02 mm to 1.02 ± 0.021 mm) (Figure 3B). The average root diameter also varied significantly among inoculated wheat treatments. The maximum root diameter due to inoculation was observed in HD2687 (173.37%), followed by HD3086 (126.87%) and HD2380 (50.06%). In comparison, the salinity-tolerant wheat cultivars, K65 and KRL210, showed significantly less improvement in root diameter upon halophilic archaeal inoculation (20.78% and 12.89%, respectively). The overall effect of halophilic archaeal inoculation seems to be higher in salinity-susceptible wheat varieties compared to tolerant varieties.

#### 3.3.3. Grain yield per spike and per plant

Inoculation with halophilic archaea resulted in a significant increase in both grain yield per spike and grain yield per plant across all wheat cultivars compared to their respective untreated controls (Table 3). The number of grains per spike ranged from 51.67 ± 1.15 to 58.00 ± 2.01 in the inoculated wheat cultivars, while it ranged from 44.00 ± 1.00 to 52.00 ± 1.01 in the untreated controls. The wheat cultivars HD2687 (20.00%) and HD3086 (19.69%) showed the maximum percentage increase in grain per spike due to inoculation, which was statistically similar to each other. These values were significantly higher than the percentage increase in grain yield per spike observed in HD2380 (13.98%), K65 (11.40%), and KRL210 (7.69%) compared to their respective untreated controls (Table 3).

Similarly, the grain yield per plant also exhibited a significant increase as a result of inoculation with *H. pelagica* CDK2 in various wheat cultivars compared to their respective untreated controls. The grain yield per plant in the inoculated cultivars ranged from 3.68 g to 5.21 g, while it ranged from 2.70 g to 3.47 g in the untreated treatments (Table 3). The increase in grain yield showed significant variation among different wheat cultivars. The wheat cultivar K65 demonstrated the highest percentage increase in grain yield (50.28%), followed by KRL210 (47.91%), HD2687 (41.85%), HD3086 (38.48%), and HD2380 (30.03%). It is worth noting that the salinity-tolerant varieties of wheat displayed a stronger response to inoculation with halophilic archaea in terms of grain yield per plant (Table 3).

#### 3.3.4. Effect of *H. pelagica* CDK2 inoculation on biochemical growth parameters and osmolyte content of wheat cultivars

##### 3.3.4.1. Total shoot protein and leaf chlorophyll content

**Figure 4:**
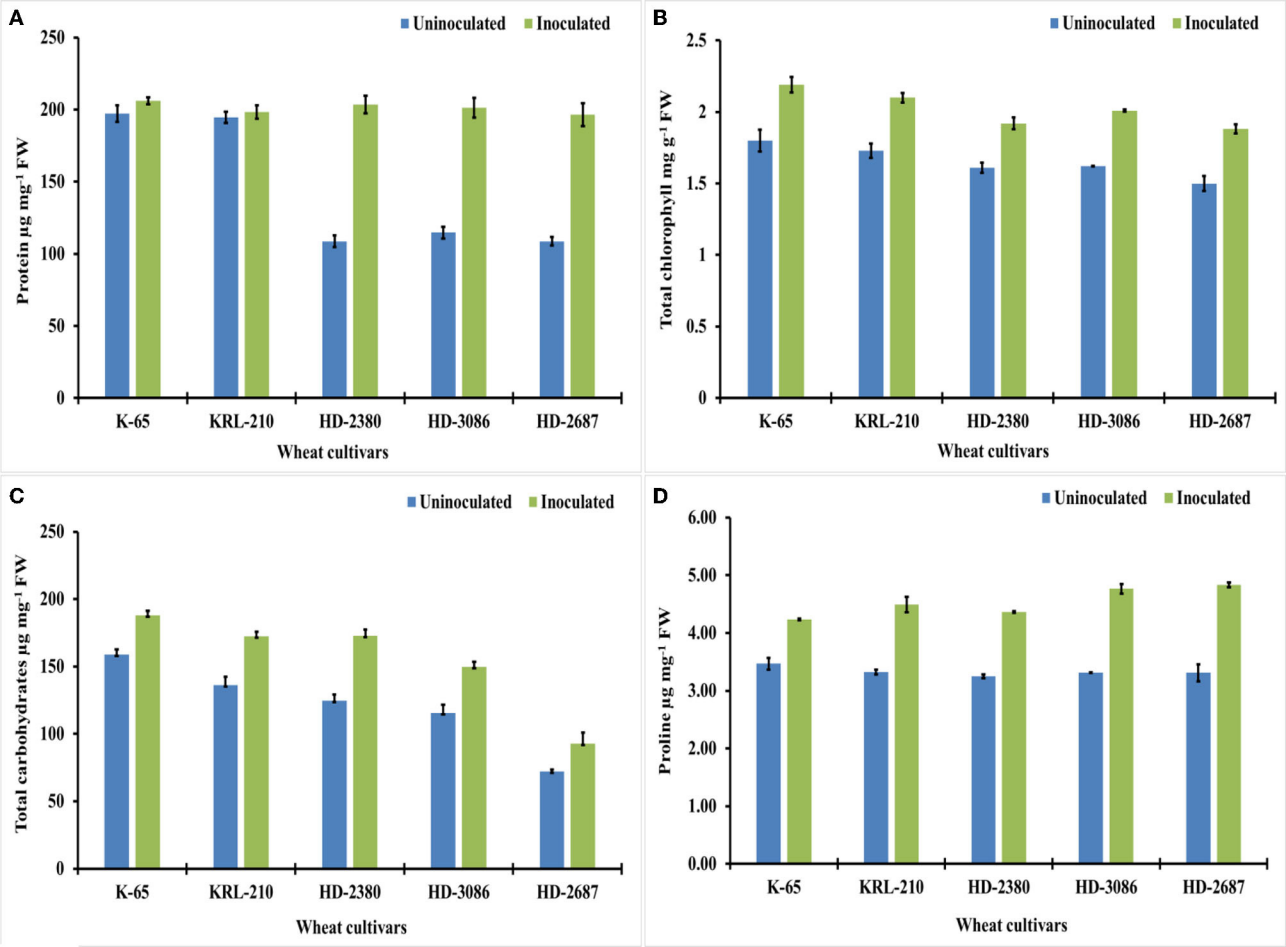
Biochemical growth parameters and osmolyte content of the wheat cultivars (pot experiment): **(A)** protein (μg mg-1 FW*), **(B)** chlorophyll (μg g-1 FW) **(C)** total carbohydrates (μg mg-1 FW), and **(D)** proline content of different wheat cultivars as influenced by archaeal inoculation (LSD *p* ≤ 0.05 for protein: Factor A (Cultivars): 6.333, Factor B (Treatments): 4.007, Interaction AxB: 8.968; LSD *p* ≤ 0.05 for chlorophyll: Factor A (Cultivars): 0.047, Factor B (Treatments): 0.034, Interaction AxB: 0.073, LSD *p* ≤ 0.05 for total carbohydrates: Factor A (Cultivars): 6.097, Factor B (Treatments): 3.856, Interaction AxB: 8.613 and LSD *p* ≤ 0.05 for proline content: Factor A (Cultivars): 0.085, Factor B (Treatments): 0.056, Interaction AxB: 0.125; error bar represents standard deviation). *FW, fresh weight.

The inoculation of wheat cultivars with *H. pelagica* CDK2 resulted in a significant increase in wheat shoot protein content across all cultivars compared to their respective untreated controls. Among the different inoculated treatments of wheat cultivars, the total shoot protein content ranged from 201.27 ± 6.92 to 205.92 ± 2.39 μg mg^−1^ fresh weight (FW) (Figure 4A) and was statistically similar. The wheat cultivars HD2687 (44.63%) and HD2380 (43.01%) exhibited the highest percentage increase in protein content due to inoculation, which was statistically similar to each other. HD3086 showed a significant increase of 29.37% in total shoot protein. However, the salinity-tolerant wheat cultivars, KRL210 and K65, demonstrated significantly lower percentage increases in total shoot protein, with values of 1.86% and 4.26%, respectively (Figure 4A).

In addition, the chlorophyll content in different inoculated wheat cultivars also showed a significant increase compared to the untreated controls (Figure 4B). The overall increase in chlorophyll content ranged from 16.14% to 23.72% due to inoculation with halophilic archaea. The salt-susceptible wheat cultivar HD2687 displayed the maximum response to inoculation, with a 20.12% increase in chlorophyll content. This increase was significantly higher than the increases observed in HD3086 (19.40%), K65 (17.80%), KRL210 (17.61%), and HD2380 (16.14%).

##### 3.3.4.2. Total carbohydrate and proline content

Treatment with *H. pelagica* CDK2 resulted in a significant increase in the total carbohydrate content of all wheat cultivars compared to their respective untreated varieties. The total carbohydrate content ranged from 92.88 ± 8.16 to 188.01 ± 3.46 μg mg^−1^ FW in the treated cultivars, which was significantly higher than the total carbohydrates ranging from 72.23 ± 1.11 to 159.0 ± 3.65 μg mg^−1^ FW in the un-inoculated treatments (Figure 4C). Among the cultivars, HD2380 showed the highest increase in total carbohydrate content (27.85%) after treatment with halophilic archaea, followed by HD3086 (22.85%), which was statistically similar to HD2687 (22.23%). KRL210 and K65 demonstrated increases of 20.93% and 15.42%, respectively. The differences in the percentage increase in total carbohydrates between HD3086, HD2687, and KRL210 were not statistically significant.

Inoculation with *H. pelagica* CDK2 resulted in a significant increase in proline content in different wheat cultivars, ranging from 18.11% to 31.51%. The proline content in un-inoculated wheat cultivars ranged from 3.25 ± 0.032 to 3.47 ± 0.103 μg g^−1^ FW, and a significant increase was observed in the respective inoculated wheat cultivars, ranging from 4.23 ± 0.011 to 4.83 ± 0.043 μg g^−1^ FW (Figure 4D). The highest percentage increase in proline content was observed in HD2687 (31.51%), followed by HD3086 (30.47%), KRL210 (25.97%), and HD2380 (25.49%), and the lowest increase was seen in K65 (18.11%).

#### 3.3.5. Impact of halophilic archaea inoculation on antioxidant enzyme activity

Seed treatment with halophilic archaea resulted in a significant reduction in antioxidant enzyme activity in all wheat cultivars. The inoculated wheat cultivars showed a decrease in ascorbate peroxidase activity ranging from 20.0% to 57.43%, while the reduction in superoxide dismutase activity ranged from 59.22% to 75.99%. The decrease in ascorbate peroxidase activity was statistically similar among the susceptible cultivars HD2687 (57.43%), HD2380 (56.08%), and HD3086 (55.17%) but significantly higher than the reduction observed in the salinity-tolerant wheat cultivars, KRL210 (23.33%) and K65 (20.17%). The highest percentage reduction in SOD activity was observed in wheat cultivars HD3086 (75.99%), followed by HD2687 (75.82%), HD2380 (68.55%), KRL210 (60.16%), and K65 (59.22%) (Table 4). Catalase activity also exhibited a significant reduction in susceptible wheat cultivars (HD2380: 61.14%, HD2687: 52.86%, and HD3086: 49.89%) compared to tolerant ones (K65: 32.50% and KRL210: 29.88%) when compared to their respective un-inoculated treatments (Table 3). The overall impact of halophilic archaeal inoculation was more pronounced in susceptible wheat cultivars than in tolerant ones. This suggests that the inoculation of halophilic archaea *H. pelagica* CDK2 has assisted wheat cultivars in mitigating salinity stress.

**Table 4:** Quantification of antioxidant enzymes in different wheat cultivars as influenced by inoculation of *H. pelagica* CDK2.

**Cultivars**	**Ascorbate peroxidase**		**SOD**		**Catalase**	
	**UI**	**I**	**UI**	**I**	**UI**	**I**
K65	14.01 ± 0.35	8.10 ± 0.33	32.5 ± 1.11	13.2 ± 0.035	10.42 ± 0.018	7.04 ± 0.21
KRL210	12 ± 0.01	9.20 ± 0.17	33.0 ± 0.18	13.1 ± 0.02	10.70 ± 0.04	7.51 ± 0.10
HD2380	17.53 ± 0.69	7.70 ± 0.09	41.2 ± 1.07	12.96 ± 0.53	15.27 ± 0.02	5.93 ± 0.18
HD3086	16.73 ± 0.61	7.50 ± 0.26	43.0 ± 1.17	10.1 ± 0.15	15.03 ± 0.31	7.53 ± 0.04
HD2687	18.17 ± 0.03	7.73 ± 0.25	41.2 ± 0.038	10.0 ± 0.16	16.13 ± 0.45	7.600 ± 0.29
LSD ≤ 0.05						
Cultivars A	0.406		0.734		0.26	
Treatment B	0.258		0.464		0.169	
Interaction (AxB)	0.574	0.1036	0.377

^*^Ascorbate peroxidase (mM g^−1^ FW min^−1^); SOD: superoxide dismutase (U g^−1^ FW min^−1^); Catalase (U g^−1^ FW min^−1^); U, unit activity; FW, fresh weight; ^**^UI, un-inoculated control; I, inoculated treatment.

## 4. Discussion

A variety of cultural and metagenomic studies across the world have changed the notion that archaea inhabit only niches with extreme physiological conditions. Furthermore, they have been discovered in normal soil conditions as well as in the rhizospheres of many crops, where they help improve plant growth (Jung et al., 2020) and form an important part of soil microbiomes, phytobiomes, and plant-associated ecosystems (Gubry-Rangin et al., 2010; Yadav et al., 2019; Taffner et al., 2020; Zhang et al., 2020; Wicaksono et al., 2022). Seasonal population dynamics of halophilic archaea inhabiting the rhizosphere of wild vegetation growing in the saline Indian desert were previously reported (Yadav et al., 2019). There is evidence that rhizospheric archaea do play an important role in the growth variety of crops, such as *Zea mays, Oryza sativa, Lycopersicon esculentus*, and *Coffea arabica*, in arid and semi-arid regions (Alori et al., 2020). However, to date, no significant reports are available in the literature indicating the direct role of rhizosphere-dwelling halophilic archaea in alleviating the harmful effects of salinity in crop plants. This study investigated the plant growth-promoting characteristics of 28 different halophilic rhizosphere-dwelling archaea and evaluated the efficacy of selected isolates in promoting the growth of wheat under salinity stress. In their respective HPS, HKS, and HZS modified media, a total of 17, 21, and 6 halophilic archaeal isolates solubilized the insoluble source of P, K, and Zn, respectively, and significantly lowered the pH of medium (up to 3.6) (Figure 1). Our previous study reported that halophilic archaea solubilizing P in different sources (rock phosphate, hydroxyapatite, and tricalcium phosphate) lowered the pH of growth media, and the lowering of pH was attributed to the production of organic acids (gluconic, citric, fumaric, succinic, propionic, formic, and tartaric acid) (Yadav et al., 2015). Hence, it can be stated that the mechanism of P solubilization by halophilic archaea is similar to that of bacteria and is accompanied by a lowering of pH due to the production of organic acids. Because no significant information is available regarding the mechanisms of K and Zn solubilization by archaea, it is possible that the lowering of pH in growing media suggests a similar process. It has been reported that most of the prokaryotes lower the pH of the growth medium by the secretion of various organic acids and/or by proton extrusion (Illmer and Schinner, 1995). In a study, Siles et al. (2022) found that organic P hydrolyzing alkaline phosphatases, *Pho*D and *Pho*X, were expressed in archaea (Euryarchaeota) isolated from arable, forest, and grassland soil, indicating that P hydrolyzes around plants.

Bacteria in the plant rhizosphere produce indole acetic acid (IAA), a key plant growth hormone that regulates cell division, cell expansion, root initiation, and lateral root formation (Goswami et al., 2014; Khatoon et al., 2020). Exogenous production of IAA is not restricted to bacteria as archaea have also been found capable of producing IAA. IAA production was observed in 17 halophilic archaeal isolates, with concentrations ranging from 17.30 to 49.3 μg ml^−1^ IAA (Figure 1C). *Halogeometricum rufum* IARI-WRAK7 produced the significantly highest quantity of IAA (49.3 μg ml^−1^) as compared to other isolates. The first report on the production of IAA by archaea was reported in *Sulfolobus acidocaldarius* (White, 1987). Later, biosynthesis of IAA was also reported in the hyperthermophilic archaeon *Ferroglobus placidus* during the anaerobic degradation of tryptophan (Aklujkar et al., 2014). A metagenomic study revealed genetic evidence for auxin biosynthesis in archaea associated with bog vegetation, which supports archaea's plant growth-promoting activity (Taffner et al., 2018). IAA production and the solubilization of P, K, and Zn by rhizosphere-dwelling halophilic archaea indicate their crucial role in ensuring that plant nutrients are available in high-salinity ecosystems. This is an important paradigm shift in how we understand plant–microbiota interactions in hypersaline environments. For further studies, *Halolamina pelagica* CDK2 was selected from 28 halophilic archaea as it produced a significant quantity of IAA and solubilized P, K, and Zn in HRA media with EC 119.8 dSm^−1^. Most agricultural soils, however, have much lower salinity levels, and halophilic archaea must be able to grow in low-to-moderate salinity levels and be able to withstand hypo-osmotic conditions genetically and biochemically. The halophilic archaea used in this study were isolated from the rhizosphere of wild grasses, non-rhizospheric soil, sediments, and saline water bodies having different salinity levels (EC ranging from 1.19 to 106.7 dSm^−1^). Our previous study demonstrated that isolates from rhizospheric soil samples with less salinity than bulk soil, sediments, or water samples could grow in a defined medium with a NaCl concentration of 10 to 25% but were not able to produce measurable growth below 10% (Yadav et al., 2019). In the past, several methods have been developed for culturing un-culturable bacteria that mimic the physicochemical conditions of natural habitats (Vartoukian et al., 2010). The concept of using soil extract in the growth medium is one such strategy to culture bacteria (Taylor, 1951). In a study, an intensive soil extract medium was successfully used to isolate previously uncultured bacteria and new taxonomic candidates, which accounted for 49% and 55% of the total isolates examined, respectively (Nguyen et al., 2018). In this study, a modified growth medium (halophilic soil extract medium) (HSE medium) with different levels of electrical conductivity (7.13 to 30.84 dSm^−1^) was developed using soil extract to test the ability of *H. pelagica* CDK2 to solubilize nutrients (P, K, and Zn) and produce IAA. *H. pelagica* CDK2 grew in the HKS broth over a wider range of ECs (7.13 to 30.84 dSm^−1^), and its growth (protein μg ml^−1^) showed an increasing trend as the EC of the HKS broth increased (Table 2). It also produced IAA and solubilized the P, K, and Zn in HSE broth (HSE-1 to HSE-9) and different levels of EC (7.13 to 30.84 dSm^−1^) (Table 2). The study showed that the selected isolate was capable of growing and exhibiting PGP attributes in HSE broth at low, moderate, and high EC. Oren (2014) reported that halophilic archaea belonging to the class Halobacteria possess wider adaptability to salinity levels for growth (1.5 M NaCl to saturation). In another study, halophilic archaea (*Haloferax* sp. and *Halogeometricum* sp.) were isolated using low salinity (2.5% NaCl) growth media, and it was concluded that they can grow at all salinity levels that are lower than those considered minimum for halophilic archaea (9–10% w/v NaCl) (Purdy et al., 2004). With the ability to grow at a wide range of salinities, *H. pelagica* CDK2 with PGP attributes was evaluated further in plant–microbe interaction studies. To date, no significant findings are available on plant–microbe interaction studies involving archaea and/or halophilic archaea. In the soft agar (EC 8 dsm^−1^) experiment, the inoculation of the halophilic archaea *H. pelagica* CDK2 significantly reduced the germination time by 2–3 d and improved the initial vegetative growth of all the wheat cultivars as compared to un-inoculated treatment (Figure 2A). Further in a pot experiment (soil with EC 8 dSm^−1^), inoculation of *H. pelagica* CDK2 in two salt-tolerant (K65 and KRL210), and three susceptible wheat cultivars (HD2380, HD3086, and HD2687) improved the vegetative parameters and grain yield per plant (30 to 50%) of wheat as compared with the un-inoculated treatment. Several researchers have previously reported the improvement in plant growth and yield parameters of plant growth under saline conditions by inoculating with different salt-tolerant bacterial isolates such as *Azospirillum brasilense* (Nabti et al., 2010), *Pseudomonas fluorescence, Bacillus pumilus*, and *Exiguobacterium aurantiacum* (Kakar et al., 2016; Sardar et al., 2023). A significant increase in wheat shoot protein content was observed across different salinity-susceptible wheat cultivars compared to salinity-tolerant wheat cultivars (1.86 to 4.26%) and un-inoculated treatments. The protein content ranged from 44.63% to 29.37% (Figure 4A). Treatment of salinity-stressed wheat plants with halotolerant *Bacillus safensis, B. pumilus*, and *Zhihengliuella halotolerant* isolated from halophytic range land plants increased leaf crude protein by 30%, water-soluble sugar content by 34%, and metabolic energy by 37% (Amini et al., 2021). Rajput et al. (2018) and Nawaz et al. (2020) both reported a significant improvement in total sugar content, protein, and chlorophyll synthesis due to the inoculation of halotolerant PGPR. After inoculation with *H. pelagica* CDK2, a significant increase in the total carbohydrate content of all wheat cultivars was observed compared to their respective un-inoculated wheat varieties. The total carbohydrate content ranged from 92.88 ± 8.16 to 188.01 ± 3.46 μg mg^−1^ FW in treated cultivars (Figure 4). Inoculation of halotolerant plant growth-promoting microbes is known to alleviate salinity stress in plants by modulating membrane integrity, proline accumulation, and reactive oxygen species scavenging and activating antioxidant mechanism (Sandhya et al., 2010; Upadhyay et al., 2012; Yogendra et al., 2015; Etesami and Noori, 2019). In this study, inoculation of halophilic archaea significantly reduced the antioxidant enzyme activity (ascorbate peroxidase, superoxide dismutase, and catalase) and improved the proline accumulation in all the wheat cultivars (Table 3 and Figure 4). Proline protects plant cells from osmotic stress damage and does not interfere with cellular machinery (Iqbal and Nazar, 2015). An increase in proline accumulation in different crops grown under salt stress due to the inoculation of halotolerant plant growth-promoting bacteria has been reported by various workers (Nawaz et al., 2020; Amini et al., 2021). This is the first report of halophilic archaea inoculating plants under salt stress and resulting in a reduction in antioxidant enzyme activity and an improvement in proline accumulation. No significant reports are available in the literature indicating this.

Archaea are still an under-detected and little-studied part of the plant rhizosphere, and their contributions to plants' health remain mostly unknown. Our data provide the first evidence of the importance of halophilic archaea as a functional component of the plant rhizosphere. In a previous study, the presence of certain plant growth-promoting and salinity resistance genes in *H. pelagica* CDK2 was reported through genome sequencing (Gaba et al., 2017); accession number: LGUC00000000) and transcriptome analysis (unpublished report; NCBI Accession number SRX13131461 and SRX13131462). The comprehensive analysis of the genome, genes, and pathways of halophilic archaea *Halolamina pelagica* CDK2 revealed the presence of genes for phosphate uptake and metabolism (polyphosphate kinase, alkaline phosphatase, phosphate ABC transporter, phosphate transport system regulatory protein, and pyrophosphate kinase), osmolyte biosynthesis (trehalose synthase and trehalose phosphatase), and antioxidant enzymes (superoxide dismutase and catalase peroxidases; Gaba et al., 2022). Through plant–microbe interaction studies, the genomic insights obtained in the previous report were corroborated. Inoculation of halophilic archaeon *H. pelagica* CDK2 showed greater potential in improving the growth and yield of susceptible wheat cultivars as compared to tolerant ones and decreased the activity of antioxidant enzymes. Hence, the inoculation alleviated the harmful effects of salinity on plants, allowing them to grow more efficiently. More efforts are needed to cultivate plant-associated archaea and to learn more about plant-associated archaeal diversity.

## 5. Conclusion

The potential of halophilic archaea isolated from the rhizosphere of wild vegetation in improving wheat growth by alleviating the effects of high salinity was investigated in this study. This is the first report highlighting the prospects of using rhizosphere-dwelling halophilic archaea in alleviating salinity-associated abiotic stress in wheat. The plant growth-promoting halophilic archaeon *Halolamina pelagica* CDK2 demonstrated tremendous potential not only in improving the vegetative growth and biochemical parameters of different wheat cultivars but also in improving osmolyte levels. The use of *H. pelagica* CDK2 also helped plants in reducing the negative effects of salinity stress by significantly lowering the level of antioxidant enzymes. As a result, this halophilic archaeon can be further evaluated in field trials before being included in various biofertilizer development programs for managing abiotic stress in agriculture.

## Data availability statement

The original contributions presented in the study are included in the article/Supplementary material, further inquiries can be directed to the corresponding author.

## Author contributions

RK developed the concept underlying the manuscript and reviewed the manuscript. MN conducted the laboratory experimentation, wrote the soft agar and pot evaluation studies, and compiled the manuscript. BR and MG reviewed the results and helped in data analysis along with the literature review. All authors contributed to the article and approved the submitted version.

## References

[B1] Ábrahám C. C. László Erdei L. S. (2010). Methods for determination of proline in plants. Methods Mol. Biol. 2, 1–14. 10.1007/978-1-60761-702-0_20 20387056

[B2] Aebi H. (1984). Catalase in Vitro. Methods Enzymol. 105, 121–126. 10.1016/S0076-6879(84)05016-3 6727660

[B3] Akinola S. A. Babalola O. O. (2021). The fungal and archaeal community within plant rhizosphere: a review on their contribution to crop safety. J. Plant Nutr. 44, 600–618. 10.1080/01904167.2020.1845376

[B4] Aklujkar M. Risso C. Smith J. Beaulieu D. Dubay R. Giloteaux L.. (2014). Anaerobic degradation of aromatic amino acids by the hyperthermophilic archaeon Ferroglobus placidus. Microbiol. 160, 2694–2709. 10.1099/mic.0.083261-0 25269449

[B5] Alori E. T. Emmanuel O. C. Glick B. R. Babalola O. O. (2020). Plant–archaea relationships: a potential means to improve crop production in arid and semi-arid regions. World J. Microbiol. Biotechnol. 36, 1–10. 10.1007/s11274-020-02910-6 32772189

[B6] Amini A. Mosleh Arani A. Ghasemi S. Rad M. H. Etesami H. Shabazi Manshadi S.. (2021). Mining the rhizosphere of halophytic rangeland plants for halotolerant bacteria to improve growth and yield of salinity-stressed wheat. Plant Physiol. Biochem. 163, 139–153. 10.1016/j.plaphy.2021.03.059 33845330

[B7] Banerjee P. Prasad B. (2020). Determination of concentration of total sodium and potassium in surface and ground water using a flame photometer. Appl. Water Sci. 10, 1–7. 10.1007/s13201-020-01188-1

[B8] Blanke M. (1992). Determination of chlorophyll using DMSO. Wein-Wissenschaft 47, 32–35.

[B9] Calanca P. P. (2017). Quantification of Climate Variability, Adaptation and Mitigation for Agricultural Sustainability. Berlin: Springer.

[B10] Chaudhary P. Xu M. Ahamad L. Chaudhary A. Kumar G. Adeleke B. S.. (2023). Application of synthetic consortia for improvement of soil fertility, pollution remediation, and agricultural productivity: A review. Agronomy 13, 643. 10.3390/agronomy13030643

[B11] Dhindsa R. S. Plumb-dhindsa P. Thorpe T. A. (1981). Leaf senescence: Correlated with increased levels of membrane permeability and lipid peroxidation, and decreased levels of superoxide dismutase and catalase. J. Exp. Bot. 32, 93–101. 10.1093/jxb/32.1.933047016

[B12] Dubois M. Gilles K. Hamilton J. K. Rebers P. A. Smith F. (1951). A colorimetric method for the determination of sugars. Nature 168, 167. 10.1038/168167a0 14875032

[B13] Etesami H. Noori F. (2019). Soil salinity as a challenge for sustainable agriculture and bacterial-mediated alleviation of salinity stress in crop plants. *Saline Soil-Based Agric*. Microoorg. 2, 1–22. 10.1007/978-981-13-8335-9_1

[B14] Filek M. Zembala M. Kornaś A. Walas S. Mrowiec H. Hartikainen H.. (2010). The uptake and translocation of macro- and microelements in rape and wheat seedlings as affected by selenium supply level. Plant Soil 336, 303–312. 10.1007/s11104-010-0481-4

[B15] Gaba S. Naitam M. G. Kumari A. Medema M. H. Kaushik R. (2022). Comprehensive genome analysis of halolamina pelagica CDK2: insights into abiotic stress tolerance genes. J. Pure Appl. Microbiol. 16, 460–470. 10.22207/JPAM.16.1.44

[B16] Gaba S. Singh R. N. Labs A. Yadav A. N. (2017). Draft genome sequence of halolamina pelagica CDK2 isolated from natural salterns from rann of Kutch, Gujarat, India. Genome Announc. 5, 2–4. 10.1128/genomeA.01593-16 28183764 PMC5331504

[B17] Gill S. S. Tuteja N. (2010). Reactive oxygen species and antioxidant machinery in abiotic stress tolerance in crop plants. Plant Physiol. Biochem. 48, 909–930. 10.1016/j.plaphy.2010.08.016 20870416

[B18] Gorji T. Yildirim A. Hamzehpour N. Tanik A. Sertel E. (2020). Soil salinity analysis of Urmia Lake Basin using Landsat-8 OLI and Sentinel-2A based spectral indices and electrical conductivity measurements. Ecol. Indic. 112, 106173. 10.1016/j.ecolind.2020.106173

[B19] Goswami D. Pithwa S. Dhandhukia P. Thakker J. N. (2014). Delineating *Kocuria turfanensis* 2M4 as a credible PGPR: A novel IAA-producing bacteria isolated from saline desert. J. Plant Interact. 9, 566–576. 10.1080/17429145.2013.871650

[B20] Gubry-Rangin C. Nicol G. W. Prosser J. I. (2010). Archaea rather than bacteria control nitrification in two agricultural acidic soils. FEMS Microbiol. Ecol. 74, 566–574. 10.1111/j.1574-6941.2010.00971.x 21039653

[B21] Hagagy N. Abdel-Mawgoud M. Akhtar N. Selim S. AbdElgawad H. (2023). The new isolated Archaea strain improved grain yield, metabolism and quality of wheat plants under Co stress conditions. J. Plant Physiol. 280, 153876. 10.1016/j.jplph.2022.153876 36444822

[B22] Hu H. Natarajan V. P. Wang F. (2021). Towards enriching and isolation of uncultivated archaea from marine sediments using a refined combination of conventional microbial cultivation methods. Mar. Life Sci. Technol. 3, 231–242. 10.1007/s42995-021-00092-0 37073339 PMC10077295

[B23] Illmer P. Schinner F. (1995). Solubilization of inorganic calcium phosphates-Solubilization mechanisms. Soil Biol. Biochem. 27, 257–263. 10.1016/0038-0717(94)00190-C

[B24] Iqbal N. Nazar R. (2015). “Proline accumulation in plants: Roles in stress tolerance and plant development,” in Osmolytes and Plants Acclimation to Changing Environment: Emerging Omics Technologies, eds N. Iqbal, R. Nazar, and N. Khan (New Delhi: Springer), 1–170. 10.1007/978-81-322-2616-1

[B25] Jung J. Kim J. S. Taffner J. Berg G. Ryu C. M. (2020). Archaea, tiny helpers of land plants. Comput. Struct. Biotechnol. J. 18, 2494–2500. 10.1016/j.csbj.2020.09.005 33005311 PMC7516179

[B26] Kakar K. U. Ren X. L. Nawaz Z. Cui Z. Q. Li B. Xie G. L.. (2016). A consortium of rhizobacterial strains and biochemical growth elicitors improve cold and drought stress tolerance in rice (*Oryza sativa* L.). Plant Biol. 18, 471–483. 10.1111/plb.12427 26681628

[B27] Khatoon Z. Huang S. Rafique M. Fakhar A. Kamran M. A. Santoyo G.. (2020). Unlocking the potential of plant growth-promoting rhizobacteria on soil health and the sustainability of agricultural systems. J. Environ. Manage. 273, 111118. 10.1016/j.jenvman.2020.111118 32741760

[B28] Kumar S. Puniya A. K. Puniya M. Dagar S. S. Sirohi S. K. Singh K.. (2009). Factors affecting rumen methanogens and methane mitigation strategies. World J. Microbiol. Biotechnol. 25, 1557–1566. 10.1007/s11274-009-0041-3

[B29] Marion M. B. (1976). A rapid and sensitive method for the quantitation microgram quantities of protein utilizing the principle of protein-dye binding. Anal. Biochem. 72, 248–254. 10.1016/0003-2697(76)90527-3 942051

[B30] Nabti E. Sahnoune M. Ghoul M. Fischer D. Hofmann A. Rothballer M.. (2010). Restoration of growth of durum wheat (Triticum durum var. waha) under saline conditions due to inoculation with the rhizosphere bacterium azospirillum brasilense nh and extracts of the marine alga ulva lactuca. J. Plant Growth Regul. 29, 6–22. 10.1007/s00344-009-9107-6

[B31] Naitam M. G. Kaushik R. (2021). Archaea: an agro-ecological perspective. Curr. Microbiol. 78, 2510–2521. 10.1007/s00284-021-02537-2 34019119

[B32] Nakano Y. Asada K. (1981). Hydrogen peroxide is scavenged by ascorbate-specific peroxidase in spinach chloroplasts. Plant Cell Physiol. 22, 867–880.

[B33] Nawaz A. Shahbaz M. Asadullah M. Imran A. Marghoob M. U. Imtiaz M.. (2020). Potential of salt tolerant PGPR in growth and yield augmentation of wheat (Triticum aestivum L.) under saline conditions. Front. Microbiol. 11, 1–12. 10.3389/fmicb.2020.02019 33117299 PMC7562815

[B34] Nguyen T. M. Seo C. Ji M. Paik M.-. J. Myung S-, W. Kima J.. (2018). Effective soil extraction method for cultivating previously uncultured soil bacteria. Appl. Environ. Microbiol. 84, 1–14. 10.1128/AEM.01145-18 30291118 PMC6275337

[B35] Ondrasek G. Rathod S. Manohara K. K. Gireesh C. Anantha M. S. Sakhare A. S.. (2022). Salt stress in plants and mitigation approaches. Plants 11, 1–21. 10.3390/plants11060717 35336599 PMC8950276

[B36] Oren A. (2014). Halophilic archaea on Earth and in space: Growth and survival under extreme conditions. Philos. Trans. R. Soc. A Math. Phys. Eng. Sci. 372. 10.1098/rsta.2014.0194 25368347

[B37] Oren A. (2019). Halophilic archaea. Encycl. Microbiol. 4, 495–503. 10.1016/B978-0-12-809633-8.20800-5

[B38] Purdy K. J. Cresswell-Maynard T. D. Nedwell D. B. McGenity T. J. Grant W. D. Timmis K. N.. (2004). Isolation of haloarchaea that grow at low salinities. Environ. Microbiol. 6, 591–595. 10.1111/j.1462-2920.2004.00592.x 15142247

[B39] Rajput L. Imran A. Mubeen F. Hafeez F. Y. Road J. Section P. P.. (2018). Wheat (*Triticum aestivum* L.) growth promotion by halo-tolerant PGPR-consortium Identification of potential halo-tolerant PGPR Physiological and Biochemical Characterization Plant inoculation assays Formulation of PGPR. Soil Env. 37, 178–189. 10.25252/SE/18/61522

[B40] Rakshit A. Singh H. B. Singh A. K. Singh U. S. Fraceto L. (2020). New Frontiers in Stress Management for Durable Agriculture. Cham: Springer.

[B41] Sadaty S. A. Nazari N. (2021). Impact of land use change on the effectiveness of traditional arable land systems and environmental in Miandorood, Mazandaran, Iran. Environ. Challenges 4, 100165. 10.1016/j.envc.2021.100165

[B42] Sandhya V. Ali S. Z. Grover M. Reddy G. Venkateswarlu B. (2010). Effect of plant growth promoting Pseudomonas spp. on compatible solutes, antioxidant status and plant growth of maize under drought stress. Plant Growth Regul. 62, 21–30. 10.1007/s10725-010-9479-4

[B43] Saravanan V. S. Subramoniam S. R. Raj S. A. (2004). Assessing in vitro solubilization potential of different zinc solubilizing bacterial (ZSB) isolates. Brazilian J. Microbiol. 35, 121–125. 10.1590/S1517-83822004000100020

[B44] Sardar H. Khalid Z. Ahsan M. Naz S. Nawaz A. Ahmad R.. (2023). Enhancement of salinity stress tolerance in lettuce (*Lactuca sativa* L.) via foliar application of nitric oxide. Plants 12, 1–24. 10.3390/plants12051115 36903975 PMC10005404

[B45] Sharma V. Singh A. Sharma D. Sharma A. Phogat S. Chakraborty N.. (2021). Stress mitigation strategies of plant growth-promoting rhizobacteria: Plant growth-promoting rhizobacteria mechanisms. Plant Sci. Today 8, 25–32. 10.14719/pst.1543

[B46] Siles J. A. Starke R. Martinovic T. Parente Fernandes M. L. Orgiazzi A. Bastida F. (2022). Distribution of phosphorus cycling genes across land uses and microbial taxonomic groups based on metagenome and genome mining. Soil Biol. Biochem. 174, 108826. 10.1016/j.soilbio.2022.108826

[B47] Širić I. Eid E. M. Taher M. A. El-Morsy M. H. E. Osman H. E. M. Kumar P.. (2022). Combined use of spent mushroom substrate biochar and PGPR improves growth, yield, and biochemical response of cauliflower (*Brassica oleracea* var. *botrytis*): A preliminary study on greenhouse cultivation. J. Hortic. 8, 830. 10.3390/horticulturae8090830

[B48] Suman A. Govindasamy V. Ramakrishnan B. Aswini K. SaiPrasad J. Sharma P.. (2022). Microbial community and function-based synthetic bioinoculants: a perspective for sustainable agriculture. Front. Microbiol. 12, 1–19. 10.3389/fmicb.2021.805498 35360654 PMC8963471

[B49] Taffner J. Bergna A. Cernava T. Berg G. (2020). Tomato-associated archaea show a cultivar-specific rhizosphere effect but an unspecific transmission by seeds. Phytobiomes J. 4, 133–141. 10.1094/PBIOMES-01-20-0017-R

[B50] Taffner J. Erlacher A. Bragina A. Berg C. Moissl-Eichinger C. Berg G.. (2018). What is the role of archaea in plants? New insights from the vegetation of alpine bogs. mSphere 3, 1–14. 10.1128/mSphere.00122-18 29743201 PMC5956146

[B51] Taylor (1951). Nature of the factor in soil-extract responsible for bacterial growth-stimulation. Nature 176, 115–116. 10.1038/168115a0 14852978

[B52] Upadhyay S. K. Singh J. S. Saxena A. K. Singh D. P. (2012). Impact of PGPR inoculation on growth and antioxidant status of wheat under saline conditions. Plant Biol. 14, 605–611. 10.1111/j.1438-8677.2011.00533.x 22136617

[B53] Vartoukian S. R. Palmer R. M. Wade W. G. (2010). Strategies for culture of “unculturable” bacteria. FEMS Microbiol. Lett. 309, 1–7. 10.1111/j.1574-6968.2010.02000.x 20487025

[B54] White R. H. (1987). Indole-3-acetic acid and 2-(indol-3-ylmethyl)indol-3-yl acetic acid in the thermophilic archaebacterium Sulfolobus acidocaldarius. J. Bacteriol. 169, 5859–5860. 10.1128/jb.169.12.5859-5860.1987 3119573 PMC214188

[B55] Wicaksono W. A. Egamberdieva D. Berg C. Mora M. Kusstatscher P. (2022). Function-based rhizosphere assembly along a gradient of desiccation in the former aral sea. MSystems 7, 1–16. 10.1128/msystems.00739-22 36377901 PMC9765073

[B56] Yadav A. Verma P. Kaushik R. Singh Dhaliwal H. Kumar Saxena A. Author C.. (2017). Archaea endowed with plant growth promoting attributes. Attrib. EC Microbiol. 8, 294–298.

[B57] Yadav A. N. Gulati S. Sharma D. Singh R. N. Rajawat M. V. S. Kumar R.. (2019). Seasonal variations in culturable archaea and their plant growth promoting attributes to predict their role in establishment of vegetation in Rann of Kutch. Biologia. 74, 1031–1043. 10.2478/s11756-019-00259-2

[B58] Yadav A. N. Sharma D. Gulati S. Singh S. Dey R. Pal K. K.. (2015). Haloarchaea endowed with phosphorus solubilization attribute implicated in phosphorus cycle. Sci. Rep. 5, 1–10. 10.1038/srep12293 26216440 PMC4516986

[B59] Yogendra S. G. U. A K. S. (2015). Bacterial mediated amelioration of drought stress in drought tolerant and susceptible cultivars of rice (*Oryza sativa* L.). African J. Biotechnol. 14, 764–773. 10.5897/AJB2015.14405

[B60] Zhang M. Liwei C. Muke H. Weiqian J. Jiabao G. Yi H. (2020). Structure, assembly, and co-occurrence of archaeal communities in tree rhizosphere of the qinghai-tibetan plateau. J. Res. 5, 1–25. 10.21203/rs.3.rs-34088/v132738877

