# Supplementary material for: Rhizosphere-dwelling halophilic archaea: a potential candidate for alleviating salinity-associated stress in agriculture
Source: Front Microbiol. 2023 Jul 26;14:1212349. doi: 10.3389/fmicb.2023.1212349 (PMC10410454; doi:10.3389/fmicb.2023.1212349)
Supplement: Supplementary file 1 [file Data_Sheet_1.pdf]

Supplementary Material

Rhizosphere dwelling halophilic archaea: a potential candidate for alleviating salinity-associated stress in agriculture

Mayur G. Naitam[1], B. Ramakrishna[1], Monendra Grover[2] and Rajeev Kaushik[1]*

*** Correspondence:**
Corresponding Author
Dr. Rajeev Kaushik
Principal scientist,
Division of Microbiology,
ICAR- Indian Agricultural Research Institute
New Delhi, India.
Email: Rajeev_micro@iari.res.in
Phone No: +91-7678275896
ORCID ID: https://orcid.org/0000-0003-1441-2551

1 Supplementary Figures and Tables

Supplementary Table 1: Quantification of growth (protein) by selected halophilic archaeal isolates grown in HRA broth for 10 days.

**Halophilic Archaea**	**Protein (mg mL^{-1})**
Haladaptatus paucihalophilus IARI-ABB4	196.08 ±7.82
Haloarcula argentinensis IARI-SOAB1	216.65±2.50
Halococcus hamelinensis IARI-SNS2	271.80±4.36
Haloarcula sp. IARI-DWAK3	159.22±6.06
Natrialba sp. IARI-SGAB2	257.51±0.45
Natrinema sp. IARI-WRS9	206.08±0.73
Haloterrigena thermotolerans IARI-SNAB1	240.08±7.70
Halobacterium sp. IARI-SNS3	170.94±2.88
Natrinema pallidum IARI-WRAK8	214.08±4.57
Haloarcula tradensis IARI-WRAK3	144.37±2.29
Haloferax sp. IARI-MAAK4	235.22±0.14
Haloferax volcanii IARI-CFAB4	254.08±1.81
Halogeometricum borinquense IARI-WRAK9	255.22±7.96
Haloterrigena sp. IARI-SOAB2	153.51±3.80
Haloterrigena hispanica IARI-SGAB3	200.65±1.42
Haloferax larsenii IARI-CFAB1	195.22±1.90
Halococcus sp. IARI-BGAK2	268.94±11.52
Natronoarchaeum mannanilyticum IARI-SSAB3	210.65±5.06
Halogeometricum rufum IARI-WRAK7	226.65±8.68
Natrinema altunense IARI-WRAK5	122.37±5.28
Natronomonas pharaonis IARI-MAAB4	164.65±6.13
Halolamina pelagica IARI-CSK1	170.94±5.61
Haloferax alexandrinus IARI-MAAB1	211.51±6.40
Halopenitus persicus IARI-MAAB3	243.51±6.51
Halorubrum sp. IARI-WRAB4	209.22±0.37
Halosarcina sp. IARI-WRAB3	196.65±3.84
Halostagnicola kamekurae IARI-TWAK7	171.22±7.02
Halolamina pelagica IARI-CDK2	252.65±8.33
**$LSD_{p\leq0.01}$**	11.288

Supplementary Table 2: Qualitative estimation of P and K solubilization potential (as D/D ratio*) of selected halophilic archaeal isolates grown in HRA broth for 10 days.

**Halophilic archaea**	**Protein (mg mL^{-1})**	**D/D ratio for P-solubilization**	**D/d ratio for K-solubilization**	**D/d ratio for Zn- solubilization**	
				**ZnO**	**$ZnCO_3$**
IARI-ABB4	196.08 ±7.82	1.33	1.4	ND	ND
IARI-SOAB1	216.65±2.50	1.4	1.25	ND	ND
IARI-SNS2	271.80±4.36	ND	ND	ND	ND
IARI-DWAK3	159.22±6.06	1.33	1.31	ND	ND
IARI-SGAB2	257.51±0.45	ND	ND	ND	ND
IARI-WRS9	206.08±0.73	ND	1.38	ND	ND
IARI-SNAB1	240.08±7.70	ND	1.5	ND	ND
IARI-SNS3	170.94±2.88	1.75	1.33	ND	ND
IARI-WRAK8	214.08±4.57	ND	ND	1.25	1.25
IARI-WRAK3	144.37±2.29	1.5	1.4	ND	ND
IARI-MAAK4	235.22±0.14	ND	1.48	ND	ND
IARI-CFAB4	254.08±1.81	ND	1.39	ND	ND
IARI-WRAK9	255.22±7.96	1.6	1.46	1.18	1.20
IARI-SOAB2	153.51±3.80	ND	1.68	ND	ND
IARI-SGAB3	200.65±1.42	ND	1.31	ND	ND
IARI-CFAB1	195.22±1.90	1.5	ND	ND	ND
IARI-BGAK2	268.94±11.52	ND	ND	ND	ND
IARI-SSAB3	210.65±5.06	1.6	1.52	ND	ND
IARI-WRAK7	226.65±8.68	1.5	1.55	ND	ND
IARI-WRAK5	122.37±5.28	ND	ND	ND	ND
IARI-MAAB4	164.65±6.13	1.6	1.66	1.22	0
IARI-CSK1	170.94±5.61	1.6	ND	ND	ND
IARI-MAAB1	211.51±6.40	1.33	ND	1.20	ND
IARI-MAAB3	243.51±6.51	1.28	1.6	ND	ND
IARI-WRAB4	209.22±0.37	1.6	1.5	1.25	ND
IARI-WRAB3	196.65±3.84	1.25	1.59	ND	ND
IARI-TWAK7	171.22±7.02	1.2	1.44	ND	ND
IARI-CDK2	252.65±8.33	1.8	1.65	1.28	1.25
**$LSD_{p\leq0.01}$**	11.288				

Supplementary Table 3: Vegetative growth parameters of different wheat cultivars as influenced by inoculation of *H. pelagica* CDK2 in a soft agar medium

Cultivars	Root biomass (mg)		Shoot biomass (mg)		Root length (cm)		Shoot Height (cm)	
	UI**	I	UI	I	UI	I	UI	I
K-65	27.47±0.86	83.73±1.81	138.64±3.37	166.97±1.05	10.83±0.20	12.96±0.30	15.93±0.15	21.3±0.45
KRL-210	38.13±0.62	66.1±2.56	164.43±0.14	190.73±1.89	7.2±0.36	8.43±0.20	18.9±0.36	22.3±0.36
HD-2380	32.93±0.71	57.0±1.64	123.9±1.78	152.0±4.52	5.86±0.20	9.760.41	21.03±0.24	25.6±0.43
HD-3086	25.07±0.38	47.35±1.40	119.23±3.11	141.83±2.17	7.46±0.05	11.23±0.15	20.84±o.31	26.53±0.58
HD-2687	34.7±1.06	64.93±0.99	160.7±0.72	204.03±7.54	7.4±0.26	11.9±0.3	18.13±0.86	22.33±0.41
LSDp≤0.05								
Cultivars A	1.718		4.175		0.255		0.555	
Treatment B	1.083		2.647		0.15		0.341	
Interaction AxB	2.423		5.903		0.357		0.775	

**UI: Uninoculated control, I: Inoculated treatment
